# Spontaneously Right‐Side‐Out‐Orientated Coupling‐Driven ROS‐Sensitive Nanoparticles on Cell Membrane Inner Leaflet for Efficient Renovation in Vascular Endothelial Injury

**DOI:** 10.1002/advs.202205093

**Published:** 2023-01-26

**Authors:** Xian Qin, Li Zhu, Yuan Zhong, Yi Wang, Guicheng Wu, Juhui Qiu, Guixue Wang, Kai Qu, Kun Zhang, Wei Wu

**Affiliations:** ^1^ Key Laboratory for Biorheological Science and Technology of Ministry of Education State and Local Joint Engineering Laboratory for Vascular Implants Bioengineering College of Chongqing University Chongqing 400030 China; ^2^ Chongqing Municipality Clinical Research Center for Endocrinology and Metabolic Diseases Chongqing University Three Gorges Hospital Chongqing 404000 China; ^3^ College of Basic Medical Sciences Chongqing Medical University Chongqing 400016 China; ^4^ JinFeng Laboratory Chongqing 401329 China

**Keywords:** biomimetic cell membrane, endothelial 6injury repair, low oscillatory shear stress, right‐side‐out orientation self‐assembly, ROS sensitive

## Abstract

Biomimetic cell membrane camouflaged technology has drawn extensive attention as a feasible and efficient way to realize the biological functions of nanoparticles from the parent cells. As the burgeoning nanotherapeutic, the right‐side‐out orientation self‐assembly and pathological dependent “on‐demand” cargo release of cell membrane camouflaged nanocarriers remarkably limit further development for practical applications. In the present study, a spontaneously right‐side‐out‐orientated coupling‐driven ROS‐sensitive nanotherapeutic has been constructed for target endothelial cells (ECs) repair through the synergistic effects of spontaneously right‐side‐out‐orientated camouflaging. This condition results from the specific affinity between the intracellular domain of key transmembrane receptors band 3 on cell membrane inner leaflet and the corresponding P4.2 peptide‐modified nanoparticles without the additional coextrusion. The “on‐demand” cargo release results from the pathological ROS‐cleavable prodrug. Particularly, the red blood cell camouflaged nanotherapeutics (RBC‐LVTNPs) can enhance target drug delivery through low oscillatory shear stress (LSS) blood flow in the injured ECs lesion. Both in vitro and in vivo results collectively confirm that RBC‐LVTNPs can restore the damaged ECs and function with the recovered vascular permeability and low inflammation microenvironment. The findings provide a powerful and universal approach for developing the biomimetic cell membrane camouflaged nanotechnology.

## Graphical abstract

Spontaneously right‐side‐out‐orientated coupling‐driven ROS‐sensitive nanoparticles through the specific affinity between the intracellular domain of key transmembrane receptors band 3 on the cell membrane and the corresponding P4.2 peptide‐modified nanoparticles without the additional coextrusion, have improved for target delivery to the vascular endothelial injury and local “on‐demand” prodrug activation triggered by the pathological ROS stimulus, thereby enhancing the endothelial repair.

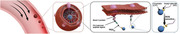

## 1 Introduction

Cell membrane camouflaging nanotechnology has attracted extensive attention, because it empowers nanoparticles to be functionalized with the unexceptionable structural and biological performances derived from the natural cell membranes,^[^ ^1^ ^]^ such as disease‐relevant targeting, long circulation, and biocompatibility.^[^ ^2^, ^3^ ^]^ However, considering the asymmetric biological properties of the cell membrane, the efficient reconstruction of the cell membrane with the right‐side‐out orientation on surface of nanoparticles remains a challenge. Accordingly, efficient spontaneous directional assembly and right‐side‐out‐orientated coating are important to the cell membrane camouflaging nanotherapeutics for further development in research and applications. Several studies have revealed that the directional assembly of cell membrane‐camouflaged nanoparticles is promoted by freeze–thaw,^[^ ^4^ ^]^ ultrasound,^[^ ^5^, ^6^ ^]^ extrusion,^[^ ^7^, ^8^ ^]^ and electric shock.^[^ ^9^, ^10^ ^]^ Unfortunately, the low success rate of spontaneous assembly and significant loss are strongly interrelated with the increased steps during the cell membrane camouflaging procedure. Therefore, it is an ideal case to improve a novel and feasible strategy for efficient cell membrane coating, especially in the absence of appropriate affinity between some cell membrane‐core pairs. A peptide ligand P4.2 (LFVRRGQPFTIILYF) with high affinity for the intracellular domain of red blood cell (RBC) membrane transmembrane protein band 3 has been designed to directionally camouflage on the core carrier through the specific binding of the intracellular fragment of protein band 3 to P4.2 peptide.^[^ ^11^, ^12^, ^13^ ^]^ This strategy is effective for the inner leaflet oriented assembly of cell membrane‐camouflaged nanoparticles.

Besides the desirable improvement in the spontaneous and oriented assembly of cell membrane camouflaged nanoparticles, the subsequent cargo release is another crucial challenge to reach the efficient therapeutic windows. In other words, the persistently strong interaction of cargo and carrier cannot meet the diversified needs at different delivery stages, i.e., the strong coupling interaction between the cell membrane and nanoparticle is beneficial for enhancing the cargo delivery and reducing the premature cargo release during blood circulation, but this process will remarkably restrict the therapeutic efficacy in the lesion. Therefore, the rational status should be that the core nanoparticles are further optimized to respond to the endogenous–exogenous stimuli for “on‐demand” drug release in the pathological lesion.^[^ ^14^ ^]^ Especially, the endogenous pathological stimulus‐triggered cargo release can be used to self‐adaptively regulate and trigger local cargo release to improve the ultimate therapeutic efficacy and minimize the toxic side effects.^[^ ^15^ ^]^ According to the pathological stimuli, the development of prodrug strategy could be available for improving the drug pharmacokinetic efficacy, particularly for targeted drug delivery.^[^ ^16^ ^]^ ROS is implicated in the pathogenesis of vascular injury and vascular endothelial cells (ECs) dysfunction.^[^ ^17^, ^18^ ^]^ ROS‐responsive prodrug has great potential applications for improving the precise treatment for vascular‐related diseases.^[^ ^19^ ^]^

ECs injury and dysfunction are early events in the development of cardiovascular disease.^[^ ^20^, ^21^, ^22^ ^]^ The maintenance and restoration of ECs integrity are essential for vascular function and the substantial recovery from ECs‐caused diseases.^[^ ^23^ ^]^ In clinic therapy, the long‐term oral administration of anti‐inflammatory and antihypertensive drugs is commonly used to indirectly restore the function of injured ECs, and this method has low bioavailability and undesirable side effects.^[^ ^24^, ^25^ ^]^ Low oscillatory shear stress (LSS) is the typical blood flow performance in the sites prone to atherosclerosis.^[^ ^26^, ^27^ ^]^ However, traditional treatments tend to ignore the blood flow, which exerts continuous fluid shear stress on the ECs lining of arteries, resulting in the injury and repair inhibition effects for ECs by the disturbed blood flow.^[^ ^28^, ^29^ ^]^ Our previous studies found that LSS could promote the uptake of RBC‐derived vesicles by ECs.^[^ ^30^ ^]^ Therefore, cell‐membrane based biomimetic nanotherapeutics is expected to enhance target delivery for the safe and efficient repair of ECs injury.

**Scheme 1:**
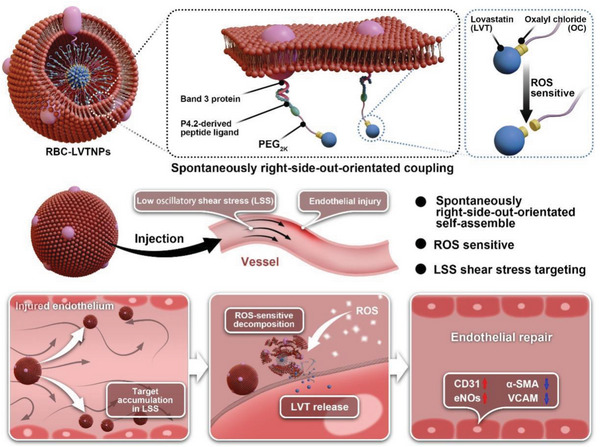
Illustrations displaying the preparation of spontaneously right‐side‐out‐orientated coupling‐driven ROS‐sensitive nanoparticles on cell membrane inner leaflet for the treatment of vascular endothelial injury.

Hence, in this study, RBC camouflaged biomimetic ROS‐sensitive nanotherapeutic was constructed for ECs injury repair. Lovastatin (LVT), which has beneficial effects to improve ECs function, increase nitric oxide production, and inhibit inflammatory cytokines,^[^ ^31^, ^32^, ^33^, ^34^ ^]^ was selected as a model drug to construct the prodrug, functionalizing with the right‐side‐out orientated coupling. Oxalyl chloride (OC) modified HO‐PEG_2K_‐Mal was used to conjugate the hydrophilic prodrug LVT and the P4.2 peptide. The resulting RBC‐coated LVTNPs (RBC‐LVTNPs) were spontaneously right‐side‐out‐orientated self‐assembled on RBC membrane inner leaflet through the specific affinity between the intracellular domain of key transmembrane receptors band 3 on the cell membrane and the corresponding P4.2 peptide‐modified nanoparticles without the additional coextrusion. It also improved target accumulation within the ECs injury and local “on‐demand” prodrug activation triggered by the pathological ROS stimulus, thereby enhancing the repair of injured ECs (**Scheme** **1**).

## 2 Result

### 2.1 Synthesis and Characterization of RBC‐LVTNPs

**Figure 1:**
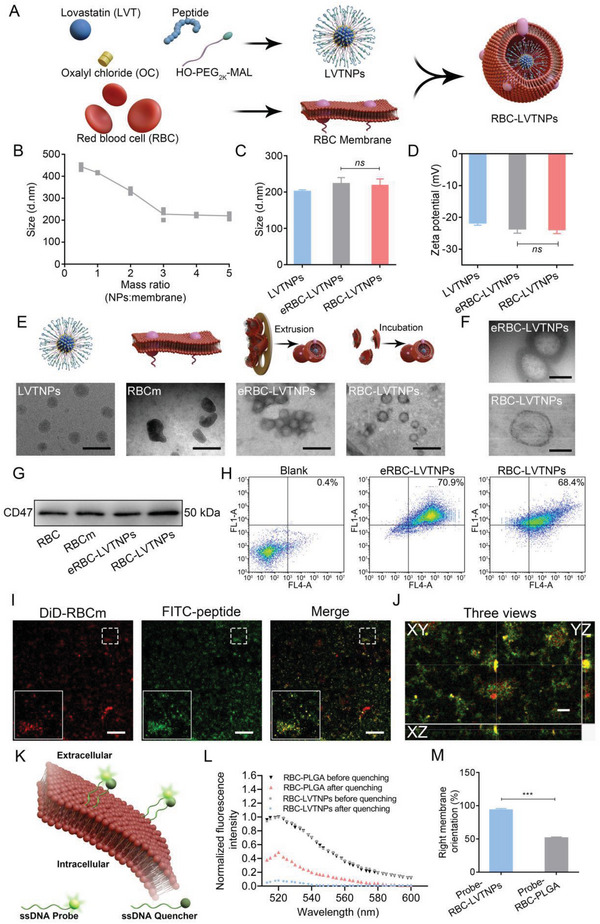
Synthesis and characterization of RBC‐LVTNPs. A) Schematic diagram of LVTNPs spontaneously right‐side‐out‐orientated coupling onto the inner leaflet of RBC membrane. B) The mass ratio of LVTNPs and RBC membrane. C) Hydrodynamic diameter and D) zeta potentials of LVTNPs, extruded RBC‐LVTNPs, and RBC‐LVTNPs (*n* = 3). E) Representative TEM images of LVTNPs, RBC membrane, extruded RBC‐LVTNPs, and RBC‐LVTNPs (*n* = 3, scale bar: 500 nm). F) TEM images of RBC‐LVTNPs and extruded RBC‐LVTNPs (scale bar: 100 nm). G) WB analysis of CD47 in RBC, RBC membrane, extruded RBC‐LVTNPs, and RBC‐LVTNPs. H) Flow cytometry analysis of DiD‐RBC membrane and FITC‐P4.2 peptide modified LVTNPs. I) Representative confocal images of DiD‐RBC membrane and FITC‐P4.2 peptide (*n* = 3, scale bar: 50 µm). J) *XYZ* axis showing the colocalization of RBC membrane and P4.2 peptide at different directions (scale bar: 10 µm). K) Schematic illustration of the cell membranes with ssDNA probes and ssDNA quencher for investigating the membrane orientation of RBC‐LVTNPs. L) Emission spectra of probe‐RBC‐LVTNPs and probe‐RBC‐PLGA in PBS before and after adding ssDNA quencher under excitation at 480 nm and M) quantifying the membrane orientation of probe‐RBC‐LVTNPs and probe‐RBC‐PLGA with FRET (*n* = 3). Significance was indicated as no significance (*ns*), or ^***^ *p* < 0.001.

For the synthesis of the ROS‐sensitive polymeric prodrug (Figure S1, Supporting Information), HO‐PEG_2K_‐Mal was acylated using OC and subsequently conjugated with LVT to obtain LVT‐PEG_2K_‐Mal. The results were validated by ^1^H NMR and ^13^C NMR (Figure S2, S3 and Table S1,S2, Supporting Information). In addition, the protein 4.2 (P4.2), a cytoskeletal protein in RBC that consists of multiple fragments, has specific affinity for the intracellular domain of protein band 3, which is distributed in RBC membranes.^[^ ^11^ ^]^ To further functionalize the polymeric prodrug to right‐side‐out orientated coupling on the inner leaflet of RBC cell membrane, we further reacted the cysteine terminated peptide sequence LFVRRGQPFTIILYC with LVT‐PEG_2K_‐Mal to obtain the functional polymeric prodrug (LVT‐PEG_2K_‐P) via Michael reaction between maleimide and sulfhydryl groups (Figure S3, Supporting Information). The results were successfully confirmed based on the matrix‐assisted laser desorption ionization time‐of‐flight mass spectrometry (MALDI‐TOF‐MS) (Figure S4, Supporting Information) and gel permeation chromatography (GPC) traces (Figure S5, Supporting Information). In addition, the critical micelle concentration for LVTNP self‐assembly was 1.8 × 10^−5^ g mL^−1^ (Figure S6, Supporting Information). Unlike the traditional methods with additional procedures to enforce the cell membrane coating on nanoparticles, the preparation of well‐defined RBC‐LVTNPs is a simple and convenient method, which involves the coincubation of the RBC membranes and LVTNP self‐assembly without any additional treatment via coextrusion or ultrasound (**Figure** **1**A).

The hydrodynamic diameter (*D*_h_) and zeta potential of RBC membranes and traditional extruded RBC membranes were examined by dynamic light scattering (DLS). As shown in Figure S7 (Supporting Information), the mean *D*_h_ values of RBC membranes and traditional extruded RBC membranes were 410 ± 3.5 and 220 ± 1.6 nm, respectively. The spontaneous coupling of RBC membranes and LVTNPs without coextrusion was further investigated. In the aqueous mixture of RBC membranes and LVTNPs, the *D*_h_ of RBC‐LVTNPs decreased when the mass ratio of LVTNPs to RBC membrane increased to 3:1, and a stable *D*_h_ plateau was maintained at a higher mass ratio. Therefore, RBC‐LVTNPs with a mass ratio at 3:1 of LVTNPs and RBC membranes were further used for all the subsequent studies (Figure 1B). The *D*_h_ and zeta potential of LVTNPs, extruded RBC membranes coated LVTNPs (eRBC‐LVTNPs), and RBC‐LVTNPs were further characterized by DLS. As shown in Figure 1C,D, and Figure S8 (Supporting Information), the mean *D*_h_ of RBC‐LVTNPs was 210 ± 2.5 nm, and the zeta potential of RBC‐LVTNPs was −26.3 mV, which was very close to that of eRBC‐LVTNPs. The polydispersity index result indicated a favorable dispersity in LVTNPs, eRBC‐LVTNPs, and RBC‐LVTNPs (Figure S9, Supporting Information).

To further confirm the morphologies, transmission electron microscopy (TEM) was introduced to visually observe LVTNPs, RBC membranes, eRBC‐LVTNPs, and RBC‐LVTNPs. The results shown in Figure 1E indicate that LVTNPs had a uniform spherical morphology that is homodispersed, while the RBC membranes exhibited an irregular morphology. Additionally, compared with the LVTNPs with uniform texture, the eRBC‐LVTNPs and RBC‐LVTNPs showed a uniform nanoscale spherical morphology with a diameter of approximately 200 nm. A significant corona layer on the surface suggested the successful RBC membrane coating on LVTNPs regardless of the presence or absence of coextrusion. However, the corona layer of RBC‐LVTNPs is much thinner than that of eRBC‐LVTNPs, and this condition can be attributed to the thriftless coating by the repeated coextrusion (Figure 1F). In addition, sodium dodecyl sulfate‐polyacrylamide gel electrophoresis (SDS‐PAGE) protein analysis showed that RBC, RBC membrane, eRBC‐LVTNPs, and RBC‐LVTNPs were highly consistent in protein bands (Figure S10, Supporting Information), and Western blot (WB) analysis indicated that the “Do not eat me” functional protein CD47 was well‐retained in RBC‐LVTNPs (Figure 1G). Moreover, LVTNPs, eRBC‐LVTNPs, and RBC‐LVTNPs sustained a relative high stability within 60 h of investigation (Figure S11, Supporting Information), indicating favorable stability for future studies.

To quantify the amount of RBC membrane and P4.2 peptide on RBC‐LVTNPs, the double‐fluorescent labeling was employed for colocalization of 1,19‐Dioctadecyl‐3,3,39,39‐tetramethylindodicarbocyanine perchlorate (DiD)‐labeled RBC membrane and fluorescein isothiocyanate (FITC)‐labeled P4.2 peptide. The RBC membrane was overlayed with the P4.2 peptide under a confocal fluorescence microscope (CLSM) (Figure 1I), and 3D views images were further processed using Imaris software to reveal the details of colocalization of RBC membrane and P4.2 peptide (Figure S12, Supporting Information). Flow cytometry analysis results further show that the double positive rates were 70.9% and 68.4% in eRBC‐LVTNPs and RBC‐LVTNPs, respectively (Figure 1H), suggesting that the protein band 3 in the RBC membrane was successfully coated on the P4.2 peptide of LVTNPs. To further confirm the “right‐side‐out” RBC membrane orientation on the nanoparticles, we checked the probe‐RBC‐LVTNPs by labeling the outer surfaces of the cell membranes, and RBC membrane camouflaged PLGA nanoparticles prepared by the traditional coextrusion method was the typical biomimetic nanoparticles used as a negative control. As shown in Figure 1K–M, 92.8% of the membranes in probe‐RBC‐LVTNPs retained a correct orientation, while 51.3% of membranes in probe‐RBC‐PLGA maintained a correct orientation by extrusion. Therefore, the majority of RBC‐LVTNPs show a correct orientation through this fabrication approach without coextruded method. These results confirmed that the well‐defined RBC‐LVTNPs with right‐side‐out‐orientated could be spontaneously and efficiently harvested using a simple and convenient mixture method.

### 2.2 Characterization of ROS‐Sensitive Drug Release and Immune‐Evasive Functions In Vitro

**Figure 2:**
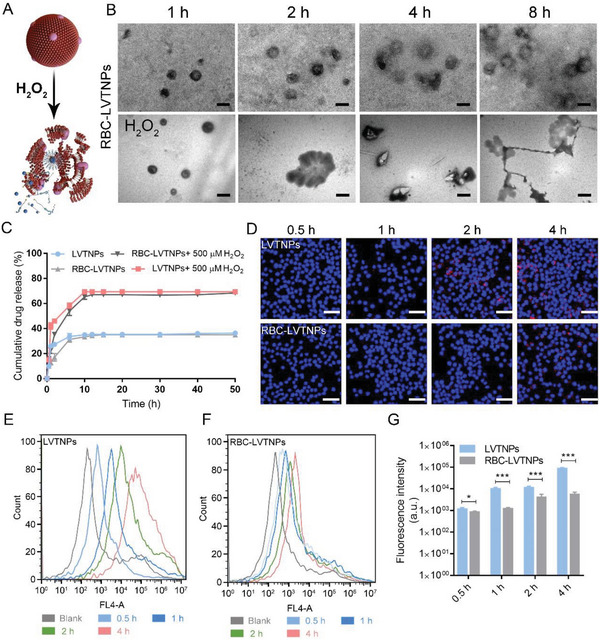
Characterization of ROS‐sensitive drug release and immune‐evasive functions in vitro. A) Schematic diagram of drug release from RBC‐LVTNPs. B) Representative TEM images of RBC‐LVTNPs with or without H_2_O_2_ at different times (scale bar: 200 nm). C) Drug release profiles of LVTNPs and RBC‐LVTNPs with or without H_2_O_2_ at different times (*n* = 3). D) Representative confocal images of the cellular uptake of LVTNPs and RBC‐LVTNPs by RAW264.7 macrophage cells (*n* = 3, scale bar: 50 µm). Flow cytometry analysis of the cellular uptake of E) LVTNPs and F) RBC‐LVTNPs by RAW264.7 macrophage cells, and G) quantification analysis of cellular uptake of LVTNPs and RBC‐LVTNPs (*n* = 3). Significance was indicated as no significance (*ns*), ^*^ *p* < 0.05, or ^***^ *p* < 0.001.

To investigate the ROS‐sensitive prodrug release, the LVTNPs and RBC‐LVTNPs were studied in the PBS with or without H_2_O_2_ (500 × 10^−6^ m). Based on the results shown in **Figure** **2**A,B, and Figure S13 (Supporting Information), compared with the stable nanoscale morphology without H_2_O_2_ stimulus, the TEM images showed that the majority of RBC‐LVTNPs was gradually destroyed under H_2_O_2_ stimulus. This phenomenon can be ascribed to the loused thermodynamic stability that resulted from the bond cleavage of OC in LVTNPs triggered by ROS stimulus. The rapid release trend was observed before the first 4 h, a relatively slow‐release stage was observed until 10 h, and the plateau was reached at 48 h. In response to ROS stimulus by using H_2_O_2_, the accumulative drug release rate of RBC‐LVTNPs increased significantly from 35.5% to 71.2% in PBS (Figure 2C), confirming that the destroyed RBC‐LVTNPs induced the accelerated prodrug activation and subsequent release from RBC‐LVTNPs. The ability of the RBC membrane coated nanoparticles to inhibit macrophages uptake and improve the long‐term blood circulation was confirmed. The cellular uptake of FITC‐labeled LVTNPs and RBC‐LVTNPs were evaluated in RAW264.7 macrophage cells. The CLSM images showed that both LVTNPs and RBC‐LVTNPs were uptake by macrophages in a time‐dependent manner. The fluorescence intensity of RBC‐LVTNPs endocytosed by macrophages significantly decreased compared with that of LVTNPs at the same time (Figure 2D). Flow cytometry analysis further confirmed that the fluorescence calculated uptake content of LVTNPs was approximately 1.2, 8.3, 2.6, and 20 times higher than that of the RBC‐LVTNPs at 0.5, 1, 2, and 4 h, respectively (Figure 2E–G). Therefore, RBC‐LVTNPs could considerably inhibit uptake by macrophages, which is highly advantageous to prolong their blood circulation time during drug delivery and reduce the undesirable clearance and augmented blood exposures.^[^ ^35^ ^]^

### 2.3 In Vitro Cytotoxicity and Blood Compatibility

Before administration treatment in vivo, the safety and excellent hemocompatibility of nanomedicine are essential prerequisites for further application. For the cell compatibility study, the cytotoxicity of free LVT, LVTNPs, eRBC‐LVTNPs, and RBC‐LVTNPs to ECs, smooth muscle cells (SMCs), and RAW264.7 macrophage cells were systematically investigated. As shown in Figure S14 (Supporting Information), after incubating the cells with free LVT, LVTNPs, eRBC‐LVTNPs, and RBC‐LVTNPs at the doses of 20, 40, 60, 80, and 100 µg mL^−1^ for 24 h, favorable cell viability was detected even when the equivalent drug concentration reached 100 µg mL^−1^, suggesting the superior cytocompatibility of LVTNPs, eRBC‐LVTNPs, and RBC‐LVTNPs. Furthermore, the blood compatibility of RBC‐LVTNPs was verified by a hemolysis test in vitro. As shown in Figure S15 (Supporting Information), no obvious hemolysis phenomenon was found in free LVT, LVTNPs, eRBC‐LVTNPs, and RBC‐LVTNPs. The hemolysis ratios of all samples were less than 5%, which was widely held as a safety standard for applications in vivo, indicating the satisfied hemolysis of free LVT, LVTNPs, eRBC‐LVTNPs, and RBC‐LVTNPs.

### 2.4 LSS Promotes ECs Repair and Target Drug Delivery In Vitro

ECs is an essential target for various drugs and gene therapy, and therapeutic nanoparticles commonly require intravascular administration, by which nanoparticles will enter the different flow stress in the bloodstream and contact with ECs.^[^ ^36^, ^37^, ^38^ ^]^ In addition, our previous research found that the LSS could selectively induce the promoted uptake of RBC‐derived vesicles by ECs.^[^ ^30^ ^]^ Therefore, the blood flow environment can be simulated in vivo by loading dynamic conditions in ECs repair and target drug delivery in vitro.

**Figure 3:**
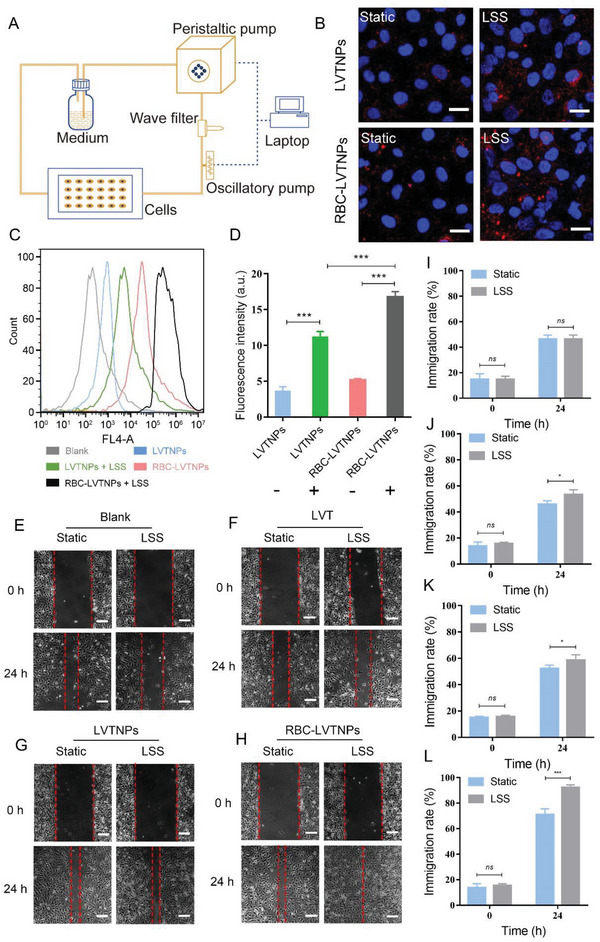
LSS promoted ECs repair and targeted drug delivery in vitro. A) Mechanical loading equipment model diagram of the parallel‐plate flow chamber. B) Representative confocal images of cellular uptake of LVTNPs and RBC‐LVTNPs by HUVEC under static and LSS condition (*n* = 3, scale bar: 50 µm). C) Flow cytometry analysis of the cellular uptake of LVTNPs and RBC‐LVTNPs, and D) quantification analysis of the cellular uptake of LVTNPs and RBC‐LVTNPs, “−” means in static condition, and “+” means in LSS condition (*n* = 3). Photographs of HUVEC migration treated with E) medium, F) free LVT, G) LVTNPs, and H) RBC‐LVTNPs at 0 and 24 h (*n* = 5, scale bar: 200 µm). Quantification analysis of HUVEC migration treated with I) medium, J) free LVT, K) LVTNPs, and L) RBC‐LVTNPs at 0 and 24 h, respectively. Significance was indicated as no significance (*ns*), ^*^ *p* < 0.05, or ^***^ *p* < 0.001.

The ECs uptake behaviors of LVTNPs and RBC‐LVTNPs were determined by CLSM and further quantified by flow cytometry under LSS. As shown in **Figure** **3**A,B and Figure S16 (Supporting Information), the CLSM images showed that RBC‐LVTNPs displayed an obviously higher uptake content than that of LVTNPs under LSS, and the flow cytometry analysis confirmed that the fluorescence intensities of LVTNPs and RBC‐LVTNPs endocytosed by ECs under LSS were 3‐fold and 3.4‐fold higher than those of LVTNPs and RBC‐LVTNPs under static, respectively (Figure 3C,D). Notably, the ECs uptake intensity of RBC‐LVTNPs was 1.3‐fold higher than that of LVTNPs under LSS. The importance of the LSS enhanced ECs uptake was further demonstrated by measuring the endothelial migration in a scratch wound assay (Figure 3E–H). The monolayer of ECs was performed uniform scratch wounds, and then incubated with the medium (blank), free LVT, LVTNPs, and RBC‐LVTNPs under static or LSS. The recovery status of the scratch gap was then recorded under a microscope at 0, 6, 12, and 24 h. The ECs migration rate of free LVT, LVTNPs, and RBC‐LVTNPs under LSS was higher than that under static at 6 and 12 h, respectively (Figure S17, Supporting Information). The immigration rate of ECs under LSS demonstrated a significant overall increase compared with that under static at 24 h, in which the EC migration rate of free LVT (55%), LVTNPs (59%), and RBC‐LVTNPs (92%) under LSS increased by 1.1, 1.2, and 1.4‐fold compared with that under static condition at 24 h, respectively (Figure 3I–L). These results confirmed that the RBC membrane decorated on LVTNPs could enhance the EC uptake of RBC‐LVTNPs, and LSS could further synergistically improve the uptake capacity of ECs, demonstrating a feasible and efficient strategy for the targeted drug delivery for ECs damage‐related diseases.

### 2.5 LSS Promoted ECs Repair and Targeted Drug Delivery In Vivo

**Figure 4:**
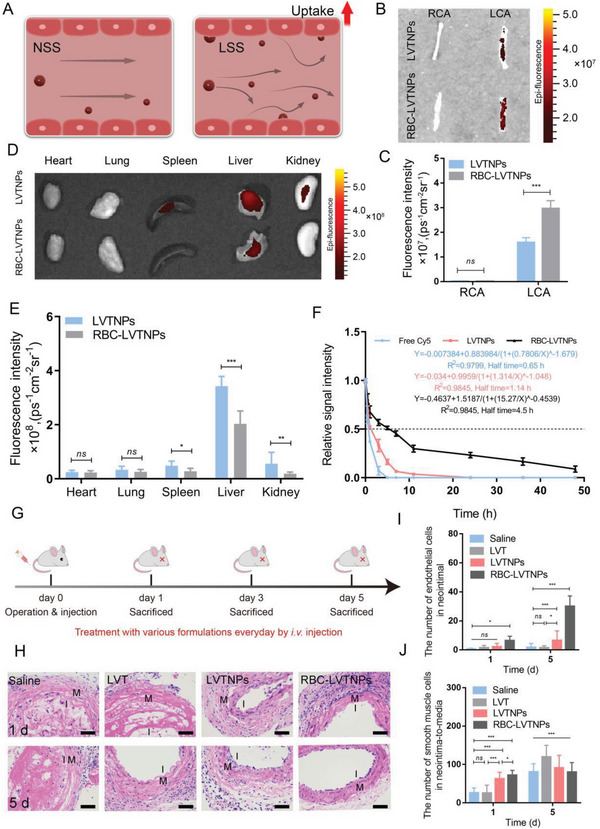
LSS promoted ECs repair and targeted drug delivery in vivo. A) Schematic diagram in which LSS promotes ECs repair in vascular compared with normal shear stress. B) The ex vivo fluorescence image and C) quantification analysis of LCA and RCA treated with LVTNPs andRBC‐LVTNPs. D) The ex vivo fluorescence image and E) quantification analysis of heart, lung, spleen, liver, and kidneys treated with LVTNPs and RBC‐LVTNPs, respectively (*n* = 5). F) Blood circulation measurements of free Cy5, LVTNPs, and RBC‐LVTNPs in vivo. G) Schematic illustration of the treatment protocols. H) H&E staining of carotid artery sections from mouse model after different treatments for 1 and 5 days; “I” represents the intima, and “M” represents the media. Images were observed at 200× magnification. Quantification analysis of I) the number of ECs in neointimal, and J) the number of SMCs in neointima‐to‐media (*n* = 8, scale bar: 50 µm). Significance was indicated as no significance (*ns*), ^*^ *p* < 0.05, or ^***^ *p* < 0.001.

The vessels of blood flow in the atherosclerotic lesions showed the typical performance of LSS.^[^ ^26^, ^27^ ^]^ Local LSS blood flow environment was created, and special blood flow environment was simulated in a pathological environment by establishing and improving a vascular guide‐wire injury model in C57BL/6 mouse and assessing the targeting ability of the RBC‐LVTNPs to the damaged ECs lesion. The surgical process is shown in Figure S18 (Supporting Information), and the surgery site of ECs injury was the LSS area (**Figure** **4**A). This process could lead to ECs damage or obvious ECs monolayer defection with pervasive vascular inflammation, new intimal formation, and eventually vascular blockage risk when the integrity of ECs restoration is lacking.^[^ ^23^ ^]^ Cyanine 5 (Cy5)‐labeled LVTNPs and RBC‐LVTNPs were intravenously injected through the tail vein, and mouse samples were euthanized after administration for 24 h. The main organs and the right and left carotid arteries (RCA and LCA) were harvested and processed *ex vivo*. The significant accumulation of RBC‐LVTNPs in the lesion of ECs injury could be clearly observed by *ex vivo* imaging. Strong fluorescence was found in the lesion of the ECs injury regions in LCA (Figure 4B,C, and Figure S19, Supporting Information). Conversely, the LVTNPs group showed relatively weak fluorescence in the ECs injury regions in LCA. The fluorescence intensity of RBC‐LVTNPs group was 3‐fold higher than that of LVTNPs. This result demonstrated that RBC‐LVTNPs could selectively accumulate in the ECs injury lesions in LCA under the stimulus of LSS. In addition, the fluorescence intensity of the liver in LVTNPs was much higher than that in RBC‐LVTNPs at 24 h post injection (Figure 4D,E), and the fluorescence of other organs was not obviously detected. Blood circulation measurement also confirmed that the RBC‐LVTNPs had much longer blood circulation time than the LVTNPs (Figure 4F). Therefore, the RBC membrane‐camouflaged nanotherapeutic could significantly reduce the undesirable accumulation of nanoparticles in the main organs, thus reducing the nonspecific toxicity and improving the biocompatibility in vivo.

Endothelial repair potency in vivo was evaluated based on the whole process, which is illustrated in Figure 4G. After each treatment for 5 days, mice were euthanized, and the blood, main organs, and carotid artery were obtained. From the results of hematoxylin‐eosin (H&E) staining of the LCA for visualizing the status of ECs injury (Figure 4H and Figure S20, Supporting Information), rare ECs were detected in the vascular intima at day 1, but the number of ECs could increase after 5 days treatment by using free LVT, LVTNPs, and RBC‐LVTNPs. The quantitative analysis of H&E staining in the areas of ECs injury demonstrated that LVTNPs and RBC‐LVTNPs treatment could slightly increase the number of the restored ECs in neointima from 2 to 7 after treatment with LVTNPs, and the number remarkably increased from 6 to 29 after treatment with RBC‐LVTNPs (Figure 4I). However, compared with the saline group, the effect of restored ECs was negligible under free LVT treatment. Moreover, considering that the increase in the migration and proliferation of SMCs may exacerbate vasoconstrictor‐mediated vascular remodeling and occlusion,^[^ ^39^, ^40^ ^]^ the number of SMCs in neointima‐to‐media under the condition of ECs injury was further measured. As shown in Figure 4J, the number of SMCs treated with RBC‐LVTNPs was obviously decreased in neointima‐to‐media under 5 days treatment, highlighting that RBC‐LVTNPs could promote an efficient therapy for promoting ECs restoration and inhibiting vascular SMCs proliferation simultaneously.

**Figure 5:**
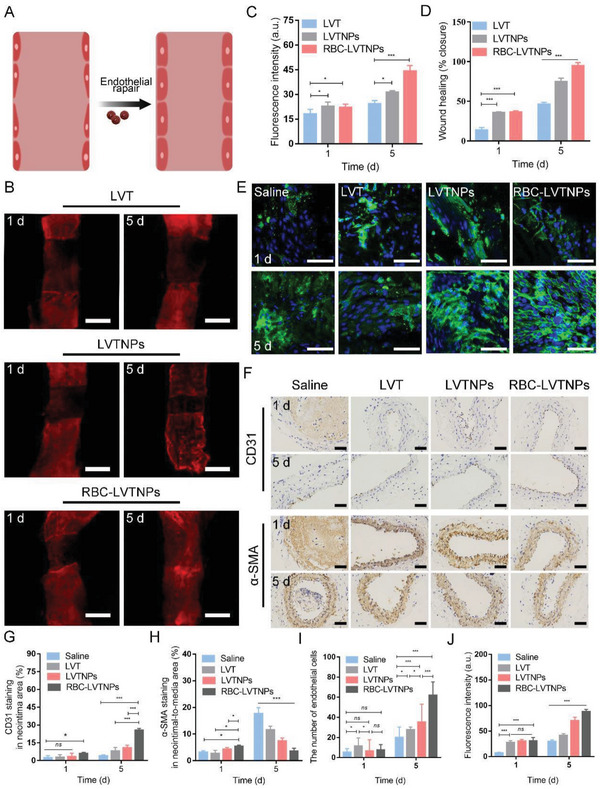
In vivo therapeutic efficacy. A) Schematic diagram before and after ECs repair. B) En face immunofluorescence images of whole‐mounted LCA after 1 and 5 days of treatment (Red: CD31, *n* = 8, scale bar: 50 µm). Quantification analysis of C) the fluorescence intensity of CD31, and D) the wound healing of ECs. E) En face immunofluorescence images of LCA after 1 and 5 days of treatment (Green: CD31, *n* = 8, scale bar: 50 µm). F) Representative immunohistochemistry staining photographs with antibodies against CD31 and *α*‐SMA after 1 and 5 days of treatment. Quantitative analysis of G) CD31 in neointimal and H) *α*‐SMA in neointima‐to‐media. Quantification analysis of I) the number of ECs, and J) the fluorescence intensity of CD31. Significance was indicated as no significance (*ns*), ^*^ *p* < 0.05, or ^***^ *p* < 0.001.

The ECs monolayer in whole‐mount LCA en face was further analyzed by CLSM (**Figure** **5**A,B). After 1 day of treatment, the wound healing ratios of the restored ECs monolayer were only 18%, 37%, and 38% after treatment with free LVT, LVTNPs, and RBC‐LVTNPs, respectively, and the wound border exhibited a regular shape and densely aligned in an orderly arrangement. After a 5 days treatment, the wound healing ratios of the restored ECs monolayer were significantly developed with values of 48%, 69%, and 98% for free LVT, LVTNPs, and RBC‐LVTNPs treatment, respectively. This finding revealed a restored ECs monolayer tendency for enhanced dynamic wound ECs closure (Figure 5C,D). In addition, one of the major initiating factors of atherosclerosis is triggered by ECs injury or denudation in atheroprone arteries.^[^ ^41^ ^]^ RBC‐LVTNPs could remarkably promote the repair of injured ECs for the pathological atherosclerosis in *apoE* ^−/−^ mice (Figure S21, Supporting Information).

To further confirm the repair of injured ECs, we investigated the en face staining and paraffin section at the LCA (Figure 5E,F). The fluorescence intensity of CD31 was almost invisible, and the nucleus of the ECs exhibited an irregular form at day 0 in all groups (Figure S22, Supporting Information). After 5 days of treatment, the RBC‐LVTNPs treatment group exhibited a confluent, homogenous, and spindle‐shaped ECs layer that covers the vascular surface, as confirmed by the immunofluorescence for CD31 (Figure 5E). Moreover, the number of ECs by RBC‐LVTNPs treatment was significantly much more than those of saline, free LVT, and LVTNPs treatment groups (Figure 5I,J). In addition, as shown in the immunohistochemistry results in Figure 5F, the ECs restoration was closely dependent on the time for treatment. After 5 days of treatment, the expression of CD31 in the neointima of RBC‐LVTNPs group reached 28.3%, which was significantly higher than that of the saline (2.1%), free LVT (7.4%), and LVTNPs (11.7%) treatment groups (Figure 5G). The *α*‐SMA staining results (Figure 5H) showed that the RBC‐LVTNPs treatment group (3.1%) had the lowest SMCs proliferation phase compared with saline (18.9%), free LVT (14.4%), and LVTNPs (8.9%) treatment groups. These results further confirmed that RBC‐LVTNPs could effectively promote ECs proliferation and inhibit the proliferation of SMCs simultaneously. This finding can be mainly attributed to the synergetic effects of the innate immune escape ability of RBC membrane and the ROS‐sensitive prodrug release for efficient and precise therapy in the injured ECs lesion.

### 2.6 Repair Mechanism of ECs Injury

**Figure 6:**
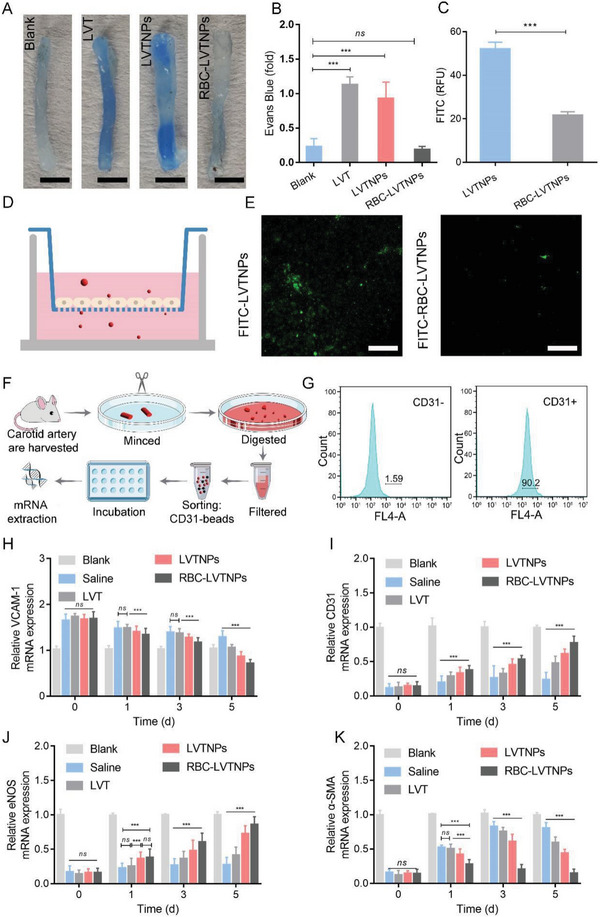
Repair mechanism of ECs injury. Representative images showing A) Evans blue dye in carotid arteries and B) quantification analysis of Evans blue dye for images (*n* = 8, scale bar: 50 µm). D) The model of Transwell assays for FITC‐LVTNPs and FITC‐RBC‐LVTNPs transmonolayer ECs. Representative confocal images of the FITC fluorescent of LVTNPs and RBC‐LVTNPs in the lower chamber (E), and the relative luminescence units of FITC‐LVTNPs and FITC‐RBC‐LVTNPs in the lower chamber (C) (*n* = 3, scale bar: 50 µm). F) Diagram of microbeads‐based protocol for EC isolation. G) Flow cytometry analysis of CD31 negative (left) and CD31 positive (right) in LCA. Quantification analysis of the mRNA expression level of H) VCAM‐1, I) CD31, J) eNOS, and K) *α*‐SMA (*n* = 15). Significance was indicated as no significance (*ns*), or ^***^ *p* < 0.001.

The restored ECs functions require morphological integrity and the dysfunction repair to promote the vascular ECs activities for maintaining the biological dynamic balance.^[^ ^42^ ^]^ Altered ECs integrity would increase vascular permeability caused by ECs disruption.^[^ ^43^ ^]^ The ECs permeability was determined by Evans blue staining and Transwell assay. As shown in **Figure** **6**A,B, the Evans blue staining results of free LVT and LVTNPs treatment groups were extravasated in dark‐blue color, suggesting high vascular permeability, while that of RBC‐LVTNPs treatment group showed mild blue color, which was comparable to that of the normal blood vessel with the uninjured vascular ECs. The ECs monolayer permeability was further confirmed by Transwell assay by using FITC‐LVTNPs and FITC‐RBC‐LVTNPs. As shown in Figure 6C–E, the FITC fluorescent intensity of RBC‐LVTNPs was 2.8‐fold higher than that of LVTNPs, indicating that the interjunction of ECs monolayer was well resealed to restoring the normal permeability after treated with RBC‐LVTNPs.

Besides the vascular permeability, ECs injury and dysfunction were remarkably influenced by inflammatory cell homing in lesion.^[^ ^44^ ^]^ As shown in Figure 6F,G, the CD31‐positive ECs were confirmed by flow cytometric analysis after anti CD31‐conjugated magnetic bead sorting. Moreover, the mRNA expression levels of VCAM‐1, TNF‐*α*, and MMP‐9, which were treated with RBC‐LVTNPs, decreased and were significantly lower than those treated with saline, free LVT, and LVTNPs. Therefore, inflammatory response was significantly weakened following treatment with RBC‐LVTNPs (Figure 6H, Figure S23A,B, Supporting Information). The mRNA expression of CD31, vWF, and VE‐cadherin, which are the key indicators of ECs surface markers, were high after treatment with the RBC‐LVTNPs, indicating the superior restoration of the ECs monolayer and connections (Figure 6I, Figure S23C,D, Supporting Information). Additionally, compared with the other treatment groups, the mRNA expression of eNOS, which is associated with vascular permeability, was upregulated after RBC‐LVTNPs treatment, demonstrating the recovered vascular permeability (Figure 6J). Among all the treatment groups, the lowest mRNA expression level of *α*‐SMA was detected after RBC‐LVTNPs treatment, indicating the positive effects on inhibiting the intimal SMCs proliferation and migration from vessel intima to the media (Figure 6K), which was consistent with the results mentioned in Figure 4J, 5H. Therefore, RBC‐LVTNPs could remarkably promote ECs proliferation and inhibit SMCs proliferation simultaneously, reduce vascular permeability and inflammation reaction, and restore the barrier and function for the efficient repairing of the injured ECs monolayer.

### 2.7 Biosafety Assessment

To assess biosafety in vivo, the potential side effects were investigated by treatment for 5 days. The complete blood count implied that RBC and white blood cells (WBC) displayed no significant change (Figure S24A–F, Supporting Information). No significant change was observed in alanine transaminase (ALT) and alkaline phosphatase (ALP) based on the liver function test. The kidney function test of blood urea nitrogen (BUN) and creatine kinase (CK) also varied within the normal range. Furthermore, the main organs of mice were stained with H&E (Figure S25, Supporting Information), indicating no obvious histological toxicity to the main organs after treatment with RBC‐LVTNPs. These results collectively suggest that RBC‐LVTNPs had a desirable therapeutic safety in vivo and were expected to be promising and feasible nanotherapeutic for EC injury repair.

## 3 Conclusion

Biomimetic membrane camouflaged nanotherapeutics has emerged as a highly promising platform to facilitate the delivery of therapeutic agents by integrating the natural biological properties of the source cells with the functional diversity of the nanomaterials. We proposed a strategy for the spontaneously directional right‐side‐out assembly of ROS‐sensitive prodrug on the inner leaflet of RBC membrane based on the intracellular domain of a membrane protein and the enhanced targeted delivery through LSS blood flow in the injured ECs lesion. For promoted therapy to repair the injured ECs, ROS‐sensitive prodrug exhibited high therapeutic efficacy and low toxicity via precise drug release at the injured ECs lesion. This progressive strategy is a remarkable development for the construction and application of biomimetic membrane camouflaged nanotherapeutic.

## 4 Experimental Section

### Materials

Peptides (LFVRRGQPFTIILYFC) was purchased from Nanjing Taiye Co. Ltd. (Nanjing, China). HO‐PEG_2K_‐Mal was purchased from Pengshuo Co. Ltd. (Shanghai, China). OC and LVT were purchased from Macklin Co. Ltd. (Shanghai, China). DiD was purchased from Biotium Inc (Fremont, USA). FITC, Cy5, and D, l‐dithiothreitol (DTT) were provided by Sigma–Aldrich (MO, USA). NAP‐5 columns were obtained from GE Healthcare (Pittsburgh, PA). 4',6‐diamidino‐2‐phenylindole (DAPI) and cell total protein extraction kits were provided by Beyotime Institute of Biotechnology (Jiangsu, China). Antibodies against CD31 (ab28364), *α*‐SMA (ab5694), and goat anti‐rabbit IgG H&L (Alexa Fluor 488) (ab150077) were obtained from Abcam. Fetal bovine serum (FBS), RPMI 1640 medium, and Dulbecco's modified Eagle's medium (DMEM) high glucose were purchased from Biological Industries (Chongqing, China). Membrane and cytosol protein extraction kit was purchased from Beyotime (Shanghai, China).

### Synthetic of ROS‐Sensitive Polymeric Prodrug (LVT‐PEG_2K_‐P)

LVT (40.4 mg, 1 mmol) and OC (63.4 mg, 5 mmol) were dissolved in 5 mL of methylene chloride (CH_2_Cl_2_), and the reaction was performed at 4 °C for 8 h with stirring. Then, the excess OC was removed via rotary evaporation. Afterward, the mixture was redissolved in CH_2_Cl_2_, and Mal‐PEG_2K_‐OH (200 mg, 1 mmol) was added. Then, the reaction mixture was stirred overnight at room temperature. The mixture was then moved to a rotary evaporation after freeze–drying, and the lyophilized products were suspended in DMSO. Then, the products were dialyzed (MWCO: 3500 Da) against deionized water to remove DMSO, and then lyophilized for 48 h to obtain the dry products. The dry products (122 mg) and P4.2 peptide (98 mg, 1 mmol) were dissolved in 5 mL of DMSO, the reaction mixture was placed in an oil bath at 40 °C for 12 h. The mixture of products was dialyzed (MWCO: 3500 Da) against deionized water to remove DMSO and lyophilized for 48 h to obtain the LVTNPs.

### Preparation of Biomimetic ROS‐Sensitive Nanoparticles (RBC‐LVTNPs)

RBC membrane vesicles were prepared as previously described.^[^ ^7^, ^30^ ^]^ RBC vesicles and LVTNPs were cocultured for 30 min at 37 °C to harvest RBC‐LVTNPs. For eRBC‐LVTNPs synthesis, RBC vesicles and LVTNPs were coextruded using an Avestin mini‐extruder (Avestin, LF‐1, Canada, 100 nm polycarbonate porous membrane) for 10 times to harvest eRBC‐LVTNPs.

### General Characterization of RBC‐LVTNPs

The LVT‐OC, LVT‐PEG_2K_‐Mal, and LVT‐PEG_2K_‐P were dissolved in DMSO‐d6 or CDCl_3_, and then 400 µL of solution was transferred into NMR tubes. The sample structures were characterized using ^1^H NMR and ^13^C NMR. The masses of LVT‐OC, LVT‐PEG_2K_‐Mal, and LVT‐PEG_2K_‐P were verified using a Bruker Autoflex MALDI‐TOF‐MS. The relative molecular weight and its distribution of the LVT‐PEG_2K_‐P were determined by using GPC. The *D*_h_ and zeta potentials were determined using a Malvern Zetasizer Nano ZS unit (Nano ZS 90, Malvern, UK) with a He–Ne laser (*λ* = 633 nm) at a scattering angle of 90° at 25 °C. The stability of RBC‐LVTNPs and eRBC‐LVTNPs dispersed in 1 × PBS and 10% serum after 60 h incubation were evaluated in terms of particle size. The membrane proteins of RBC, RBC‐LVTNPs, and eRBC‐LVTNPs were extracted using the membrane and cytosol protein extraction kit and used for SDS‐PAGE. The expression of CD47 was detected by WB). The morphologies of RBC‐LVTNPs and eRBC‐LVTNPs were observed by TEM at 200 kV (JEM‐2100F, JEOL, Japan). For the preparation of the TEM samples, RBC‐LVTNPs and eRBC‐LVTNPs suspension droplets were dripped on copper‐coated mesh grids for 2 min and rinsed in filtered 1 × PBS. RBC‐LVTNPs and eRBC‐LVTNPs on the grids were immediately fixed with 4% glutaraldehyde for 1 min, and then negatively stained with 2% (wt/vol) sodium phosphotungstate for 1 min.

### Preparation of the ssDNA Probe to RBC‐LVTNPs

ssDNA probe was prepared using a reported method with some modifications.^[^ ^45^ ^]^ In brief, the ssDNA probe (50 × 10^−6^ m) was reduced in DTT (0.1 m, 0.17 m phosphate buffer solution, pH = 8.0) for 1 h. Then, the ssDNA probe was purified using a NAP‐5 column (GE Healthcare) with PBS as the eluent. RBC membrane was incubated with the ssDNA probe at 25 × 10^−6^ m for 1 h at room temperature, and then sonicated and washed with PBS to obtain probe‐RBC. Then, the probe‐RBC coincubation LVTNPs were kept at 4 °C for 1 h to produce probe‐RBC‐LVTNPs. To obtain probe‐RBC‐PLGA, probe‐RBC and PLGA coextruded by using an Avestin mini‐extruder 10 times.

The percentage of probe‐RBC‐LVTNPs and probe‐RBC‐PLGA with a correct membrane orientation (right‐side‐out) *p* was calculated using the following equation:

(1) $$p=\left(F_{0}-F_{1}\right)/\left(F_{0}-F_{2}\right)$$

where *F_0_* is the fluorescence intensity at 520 nm that was measured at 480 nm as the excitation wavelength, *F*_1_ is the fluorescence intensity at 20 min after quenching (0.4 × 10^−6^ m), and *F*_2_ is the fluorescence intensity of background.

### In Vitro Drug Release from Nanotherapeutics

The drug release was examined in vitro by using the dialysis method.^[^ ^46^ ^]^ LVTNPs and RBC‐LVTNPs solutions (1 mg mL^−1^) were added to the dialysis bag (MWCO: 3500 Da) without or with H_2_O_2_ (500 × 10^−6^ m), respectively, and immersed in 50 mL of release medium. At predetermined time interval, 2 mL of the release medium of the dialysis was collected, and the same volume of fresh medium was added. The concentration of drug release was calculated using a UV–Vis spectrophotometer at 280 nm according to the standard curve.

### Parallel‐Plate Flow Chamber

The cultured cells were subjected to shear stress in a parallel‐plate flow chamber as described previously.^[^ ^47^ ^]^ The flow apparatus contained a peristaltic pump and an oscillatory pump (a frequency of 1 Hz and peak amplitude of 0.5 ± 4 dyn cm^−2^) for LSS. The following formula was used:

(2) $$\tau =6\;\mu {Q\;\mathrm{wh}}^{-2}$$

where *τ* is the fluid shear stress, *Q* is the flow rate, and *µ* is the dynamic viscosity of the perfusate.

### Cell Culture

HUVEC, RAW264.7 macrophage cells, and SMCs were obtained from the Cell Bank of the Chinese Academy of Science (Shanghai, China). Cells were grown in RPMI‐1640 medium or DMEM high‐glucose medium containing 10% FBS at 37 °C in an atmosphere with 5% CO_2_.

### In Vivo Toxicity Assays

The cell viability was quantified by MTS assay. HUVEC, RAW264.7 macrophage cells, and SMCs were seeded in a 96‐well plate. These cells were cultured with different concentrations of free LVT, LVTNPs, eRBC‐LVTNPs, and RBC‐LVTNPs in serum‐free medium for 24 h.

### Macrophage Uptake Study

To test the stealth ability of RBC‐LVTNPs, murine RAW 264.7 macrophage cell was chosen for cell uptake studies.^[^ ^34^ ^]^ RAW264.7 was seeded in 6‐well plates at a density of 1 × 10^5^ cells per well in 2 mL of DMEM high‐glucose medium containing 10% FBS and cultured at 37 °C with 5% CO_2_ for 24 h. Then, FITC‐labeled RBC and RBC‐LVTNPs were added to each well, and the samples were incubated for different times. For fluorescence imaging, the cells were washed three times with 1× PBS and fixed with 4% paraformaldehyde (PFA) at room temperature for 30 min. Then, the nuclei were stained with DAPI for another 15 min, and the cells were observed using CLSM (Leica).

### ECs Uptake Study

HUVEC was seeded in 6‐well plates at a density of 1 × 10^5^ cells per well in 2 mL of RPMI‐1640 medium containing 10% FBS and cultured at 37 °C with 5% CO_2_ for 24 h. Before parallel‐plate flow chamber loading, the FITC‐labeled LVTNPs and RBC‐LVTNPs were added to each well and incubated for 3 h. Then, the cells were observed by CLSM.

### Wound‐Healing Assay

HUVEC was seeded in cell climbing slices and cultured until reaching 95% confluency. Before mechanical loading, the adherent monolayer cells were scratched using 200 µL pipette tip. The scratch wounds were marked with dots by using a labeling pen and added to medium, free LVT, LVTNPs, and RBC‐LVTNPs. The cell climbing slices were placed on the parallel‐plate flow chamber and incubated with 5% CO_2_ at 37 °C for different times. Images of the scratch wounds were captured using a phase‐contrast microscope. The percentage of wound‐healing was calculated using the following formula:

(3) $$\left(a-b\right)\times a^{-1}\times 100\%$$

where *a* is the original scratch width, and *b* is the scratch width after healing.

### Flow Cytometry

A total of 1 × 10^5^ cells (HUVEC or RAW264.7) were inoculated on 6‐well plates in advance and incubated with FITC‐labeled LVTNPs and RBC‐LVTNPs. The cells were digested with trypsin, centrifuged at 150 × *g* for 5 min, and washed twice with 1× PBS. Then, a cell filter was used to uniformly disperse the cell mass. Flow cytometry data were plotted and quantified based on the mean fluorescence intensity by using the FlowJo software (Treestar, Ashland, USA)

### Hemolysis Assay

RBCs from whole blood of healthy C57BL/6 mice were collected and washed with saline for three times (150 × *g*, 10 min, 4 °C). Different groups in normal saline were incubated with RBCs suspension at 37 °C for 1 h. Pure water was used as positive control while normal saline was used as negative control. The absorbance of the supernatants collected by centrifuging at 1000 × *g* for 5 min from each group was measured at 545 nm by using a UV–Vis spectrophotometer.

The hemolytic activity (%) was calculated using the following equation:

(4) $$\mathrm{Hemolytic}\;\mathrm{activity}=\left({\mathrm{OD}}_{T}-{\mathrm{OD}}_{\mathrm{NC}}\right)/\left({\mathrm{OD}}_{\mathrm{PC}}-{\mathrm{OD}}_{\mathrm{NC}}\right)\times 100\%$$

where OD_T_ is the OD test value obtained in the presence of free LVT, LVTNPs, or RBC‐LVTNPs, OD_NC_ is the negative control, and the OD_PC_ is the positive control.

### Transwell Assays

Transwell cell migration assay was performed as previously described.^[^ ^48^ ^]^ A total of 1 × 10^5^ HUVEC in 100 µL serum free media were added into the upper chamber, and 500 µL of culture media was added to the lower well and cultured at 37 °C with 5% CO_2_ for 12 h. Then, the FITC‐labeled LVTNPs and RBC‐LVTNPs (100 µL, 100 µg mL^−1^) were added into the upper chamber. The fluorescence of the medium from the upper chamber to the lower chamber was first made into a smear, and then dried with 4% PFA for 10 min. The smear was observed using a CLSM, and the fluorescence intensity was determined using a fluorescence microplate reader (FlexStation II, NanoDrop, USA).

### Animals

C57BL/6 mice (8‐week‐old male) were obtained from Beijing HFK Bioscience Co., Ltd. (Beijing, China). The procedures were carried out based on institutional and national guidelines for the care and use of laboratory animals. All the animal care and experimental protocols were carried out with review and approval of the Institutional Animal Care and Use Committee (IACUC) of Chongqing University (IACUC Issue No.: CQU‐IACUC‐RE‐202109‐002)

### In Vivo Blood Circulation Measurement

For the study of the blood circulation of RBC‐LVTNPs, 200 µL of free Cy5, Cy5‐labeled and RBC‐LVTNPs were injected into the tail vein of mice. Blood samples were collected at 0.25, 0.5, 1, 2, 4, 8, 12, 24, 36, 48 h after injection. The blood samples were diluted with 40 µL of PBS containing 0.2 × 10^−3^ m EDTA_2K_ in 96‐well plates, and the fluorescence intensity was determined using a fluorescence microplate reader (FlexStation II, NanoDrop).

### Carotid Artery Guide‐Wire Injury Model

The carotid artery injury mouse model was developed as described previously with some modifications.^[^ ^19^ ^]^ C57BL/6 mice were fed a normal chow diet under a strict 12 h light cycle, and the mice were anesthetized via intraperitoneal injection of pentobarbital sodium (1%, 40 mg kg^−1^; Dainippon Pharmaceutical, Tokyo, Japan). The LCAs were separated using sterile surgical instruments, and the external carotid artery was ligated with an 8‐0 suture at the proximal end of the bifurcation point. Vascular clamp was used to block the blood flow of the internal carotid artery, external carotid artery, and common carotid artery. The guidewire (0.38 mm in diameter) was inserted into the LCA incision and withdrawn for 10 times with a rotating motion. After carefully removing the guidewire, the vascular clamp was released, and blood flow was restored, but the ligation of the internal and external carotid artery was maintained to create a local LSS condition. As the sham group, the contralateral RCAs were separated but not injured.

After injury, the mice were randomly divided into four groups and injected with 150 µL of the following chemicals via the tail vein every day: (a) saline, (b) free LVT, (c) LVTNPs, and (d) RBC‐LVTNPs. The injection dose was 20 mg kg^−1^, and the mice were subjected to the treatments for 5 days. After treatment, the mice were sacrificed.

The ex vivo target delivery was examined by injecting 150 µL of Cy5‐labeled LVTNPs and RBC‐LVTNPs through the tail vein. After 24 h, the LCA/RCA and organs were collected and washed three times with 1 × PBS to remove the blood. The imaging and fluorescence quantification of vessels and organs were performed using the in vivo small animal optical imaging system.

### Permeability Experiment

The permeability experiment was performed as previously described.^[^ ^49^ ^]^ Mouse samples were injected with 5 mg kg^−1^ 1% Evans blue (Sigma, USA). Then, the samples were anesthetized via intraperitoneal injection with pentobarbital sodium. Evans blue dye in the carotid artery was photographed using a digital camera (Nikon, Japan), extracted by formamide, and quantified via spectrophotometry (Thermo Fisher Scientific, USA).

### Immunofluorescence

The cells or frozen sections were fixed in 4% PFA for 15 min at room temperature. The cells or frozen sections were blocked in 5% bovine serum albumin for 1 h, and then incubated overnight with CD31 antigen at 4 °C. Then, the cells or frozen sections were washed and incubated with fluorescence‐coupled secondary antibodies for 2 h. The nuclei were stained with DAPI. The cells or frozen sections were observed by CLSM.

### Histology and Immunohistochemistry Staining

The LCA and RCA were fixed with 4% PFA for 1 h, and then prepared to paraffin sections. For histological analysis, the sections of the main organs, including heart, liver, spleen, lung, kidney, and carotid artery, were analyzed by H&E staining. For immunohistochemistry analysis, sections were incubated with antibodies, including CD31 and *α*‐SMA.

### Isolation of ECs from Carotid Artery

ECs were isolated by antibody‐coated dynabeads sorting. In brief, the carotid artery was shredded using scissors and digested with collagenase I and positive immunoselection sorted by CD31 dynabeads (Thermo Fisher Scientific).

### Total RNA Isolation and Quantitative Real‐Time PCR

The total RNA of carotid artery was extracted using RNAiso reagent (9109, Takara, CHN). Quantitative real‐time PCR was performed on Roche's LightCycler 480 real‐time PCR system. The primers for each specific gene are listed in Table S3 (Supporting Information).

### Blood and Serum Biochemistry Analysis

Blood was analyzed using an automated hematology analyzer (Sysmex KX‐21, Sysmex Co., Japan). The concentrations of RBC, WBC, ALT, ALP, BUN, and CK in blood or serum from different treatments were quantified using an automated analyzer platform (Roche Cobas C501, Roche Co., Switzerland).

### Statistical Analysis

Statistical analyses were performed using the Statistical Package for Social Sciences. Data were presented as mean ± SD. Experiments were performed with a minimum of three replications. Tukey's multiple comparison test, Mann–Whitney *U* test, and Student *t*‐test were used to identify significant differences where appropriate. Significance was indicated as no significance (*ns*), or ^*^ *p* < 0.05, ^**^ *p* < 0.01, ^***^ *p* < 0.001.

## Conflict of Interest

The authors declare no conflict of interest.

## Supporting information

Supporting Information

## Data Availability

The data that support the findings of this study are available from the corresponding author upon reasonable request.
