# Supplementary material for: Spontaneously Right‐Side‐Out‐Orientated Coupling‐Driven ROS‐Sensitive Nanoparticles on Cell Membrane Inner Leaflet for Efficient Renovation in Vascular Endothelial Injury
Source: Adv Sci (Weinh). 2023 Jan 26;10(6):2205093. doi: 10.1002/advs.202205093 (PMC9951580; doi:10.1002/advs.202205093)
Supplement: Supplementary file 1 — Supporting Information [file ADVS-10-2205093-s001.pdf]

ADVANCED SCIENCE
Open Access

Supporting Information

for *Adv. Sci.*, DOI 10.1002/advs.202205093

Spontaneously Right-Side-Out-Orientated Coupling-Driven ROS-Sensitive Nanoparticles on Cell Membrane Inner Leaflet for Efficient Renovation in Vascular Endothelial Injury

Xian Qin, Li Zhu, Yuan Zhong, Yi Wang, Guicheng Wu, Juhui Qiu, Guixue Wang, Kai Qu, Kun Zhang* and Wei Wu**

Supplementary materials for

Spontaneously Right-Side-Out-Orientated Coupling-Driven ROS-Sensitive Nanoparticles on Cell Membrane Inner Leaflet for Efficient Renovation in Vascular Endothelial Injury

Xian Qin, Li Zhu, Yuan Zhong, Yi Wang, Guicheng Wu, Juhui Qiu, Guixue Wang, Kai Qu, Kun Zhang,* and Wei Wu**

X. Qin, L. Zhu, Y. Zhong, J. Qiu, G. Wang, K. Qu, K. Zhang, W. Wu

Key Laboratory for Biorheological Science and Technology of Ministry of Education, State and Local Joint Engineering Laboratory for Vascular Implants, Bioengineering College of Chongqing University

Chongqing, 400030, China

E-mail: qukaigood@126.com (K. Qu); kunzh01@163.com (K. Zhang); david2015@cqu.edu.cn (W. Wu)

X. Qin, G. Wu, K. Qu, K. Zhang

Chongqing University Three Gorges Hospital, Chongqing Municipality Clinical Research Center for Endocrinology and Metabolic Diseases

Chongqing, 404000, China

Y. Wang

College of Basic Medical Sciences, Chongqing Medical University

Chongqing, 400016, China.

G. Wang, W. Wu

JinFeng Laboratory

Chongqing, 401329, China

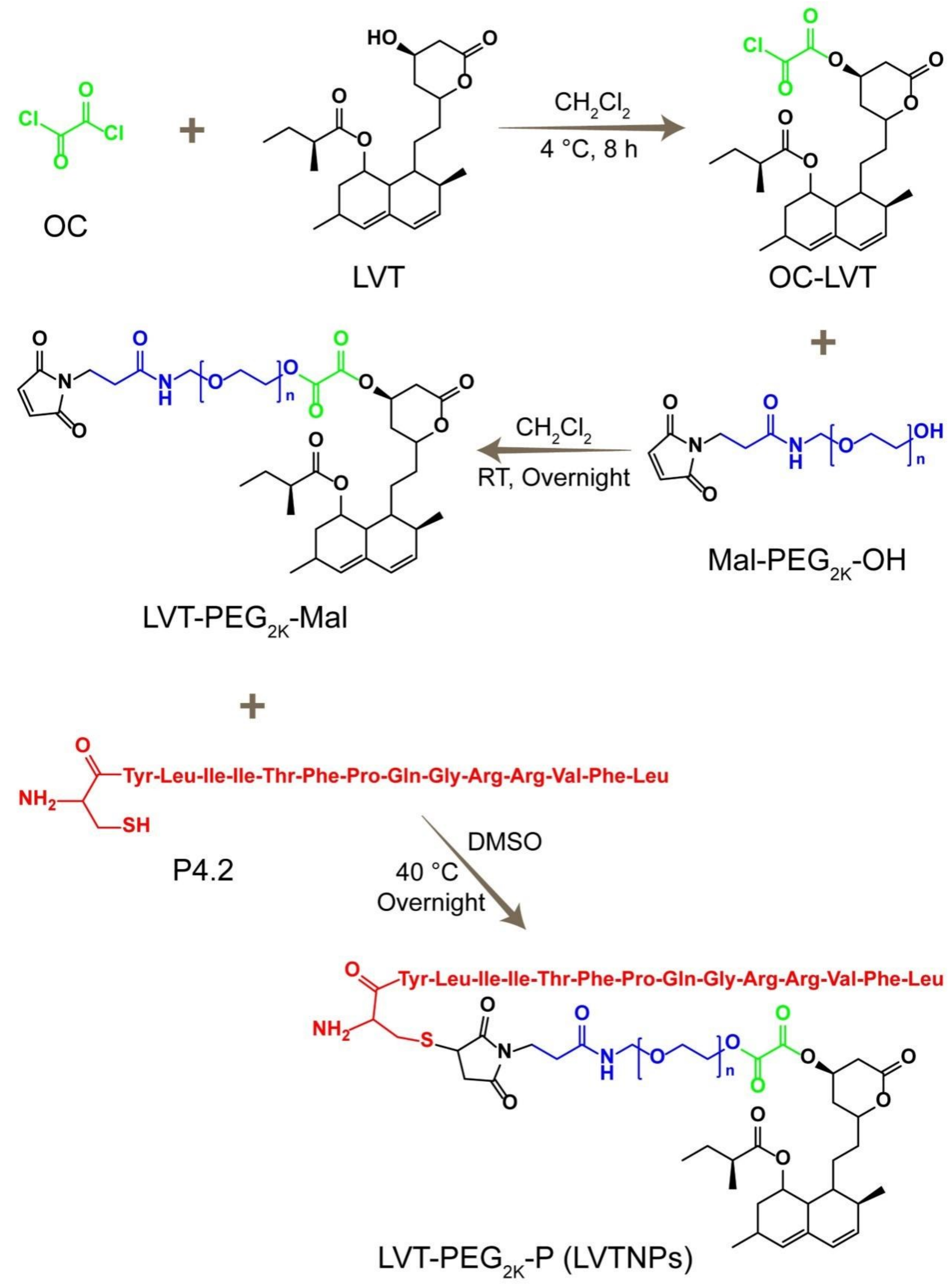

Figure S1. The synthesis of LVTNPs.

A

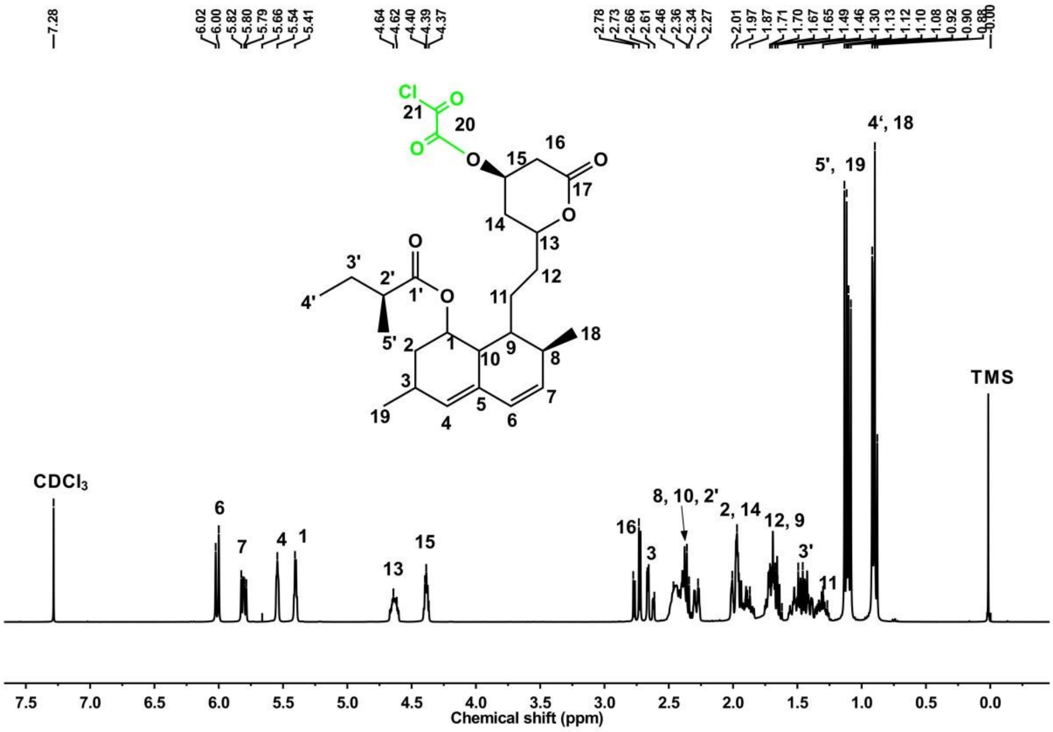

B

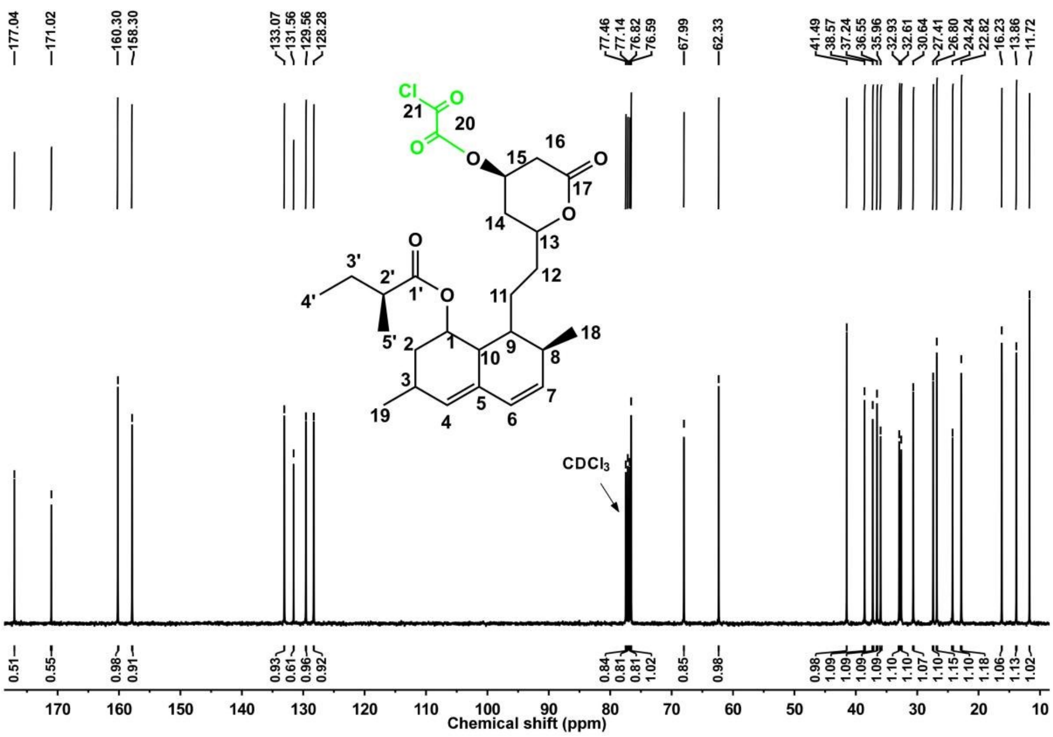

Figure S2. (A) ^{1}H NMR spectrum of LVT-OC. (B) ^{13}C NMR spectrum of LVT-OC.

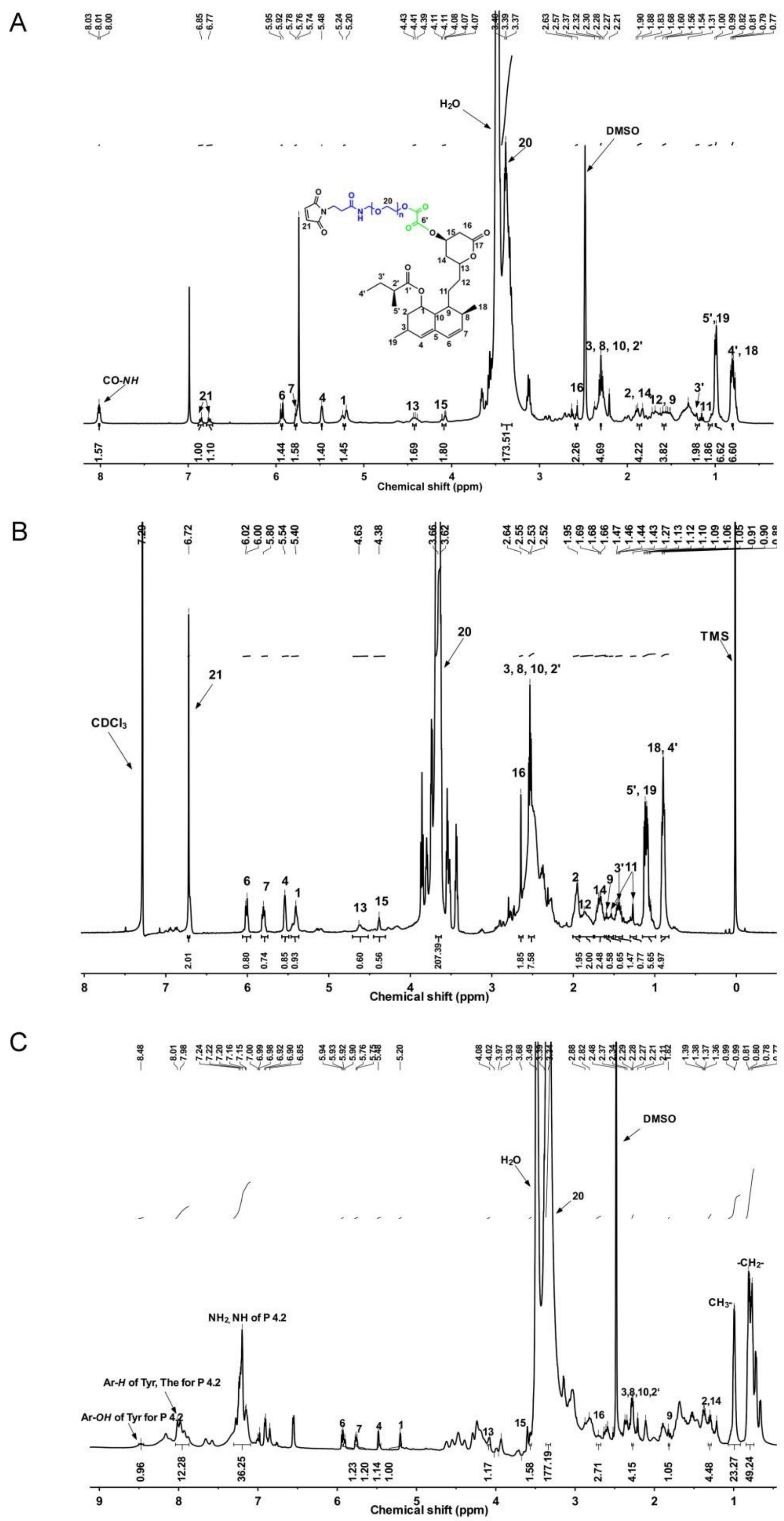

Figure S3. ^{1}H NMR spectrum of (A) LVT-PEG$_{2K}$-Mal in DMSO-*d6* and (B) LVT-PEG$_{2K}$-Mal in $CDCl_3$. (C) LVT-PEG$_{2K}$-P in DMSO-*d6*.

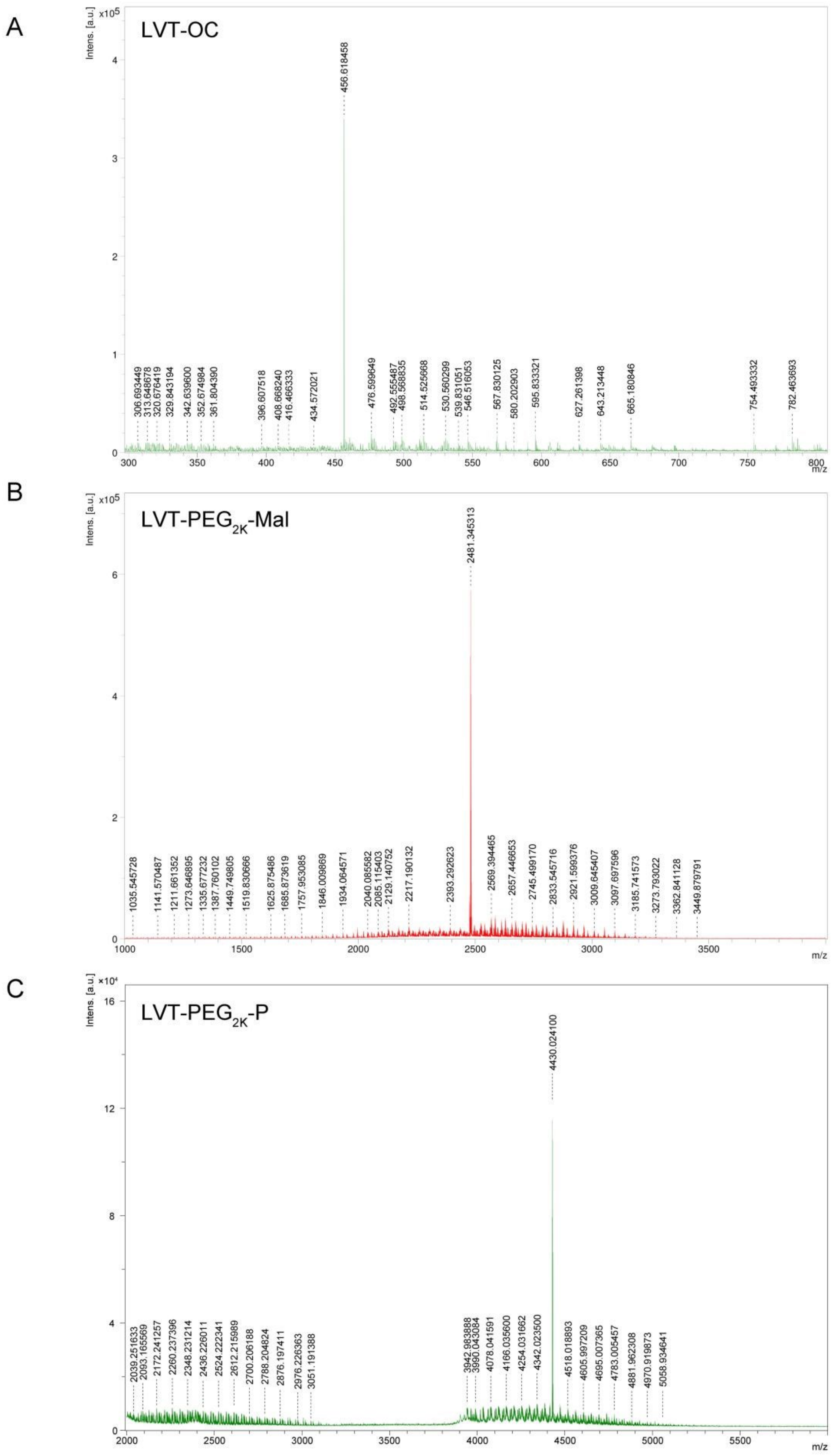

Figure S4. MALDI-TOF-MS spectra of (A) LVT-OC and (B) LVT-PEG$_{2K}$-Mal. (C) LVT-PEG$_{2K}$-P.

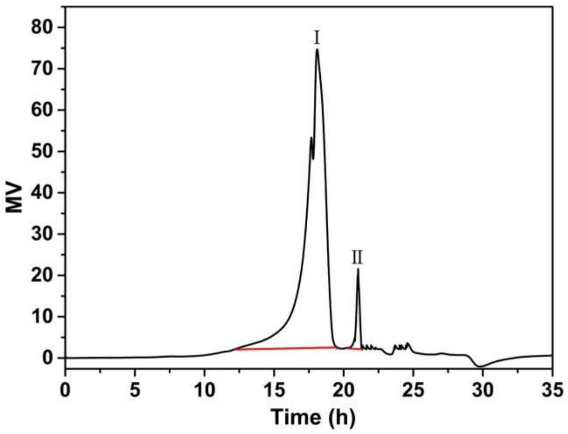

Peak No.	RT (min)	Area	Mn	Mp	Mw	PDI	Content (%)
Peak I	18.51	129.6241	4572	4363	5917	1.2942	94.16
Peak II	21.20	7.8552	1971	1871	1996	1.0127	5.84

Figure S5. GPC traces of LVT-PEG_{2K}-P.

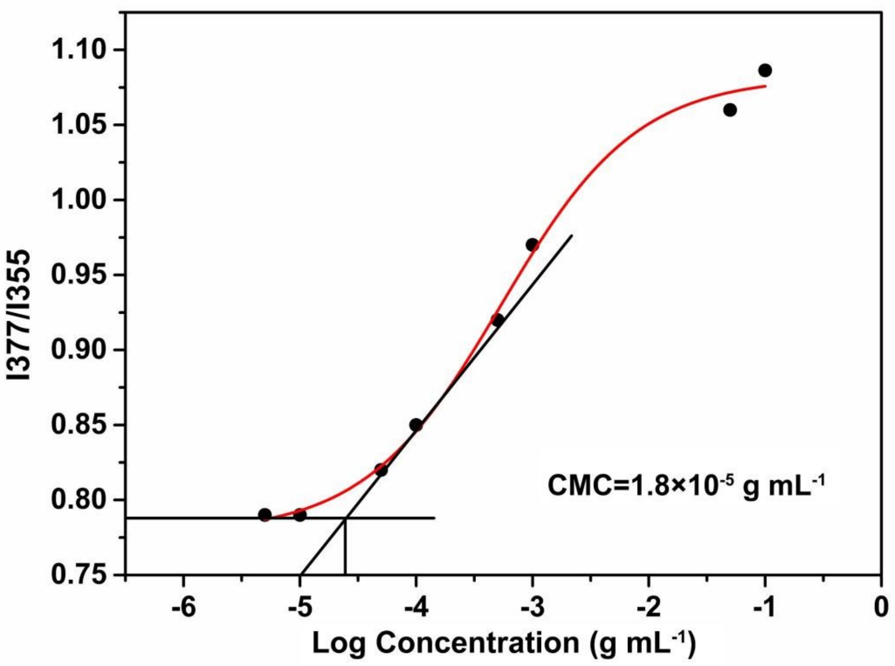

Figure S6. Critical micelle concentration of LVT-PEG_{2K}-P.

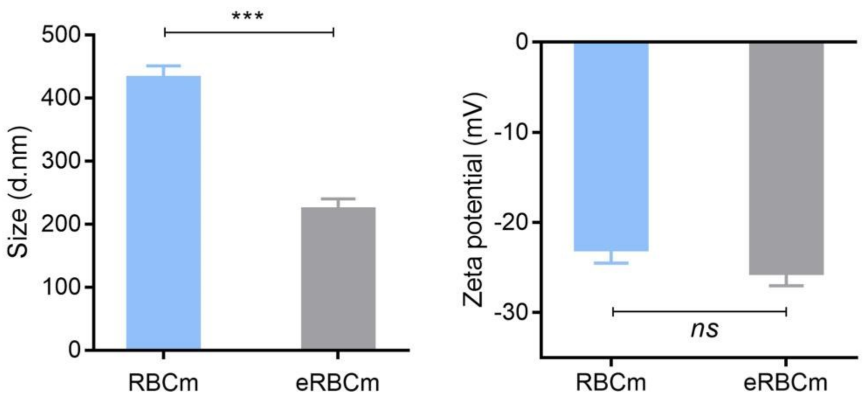

Figure S7. Hydrodynamic sizes and zeta potentials of RBC membrane and eRBC membrane (n = 3). Significance was indicated as no significance (*ns*), or $P < 0.001$ (***).

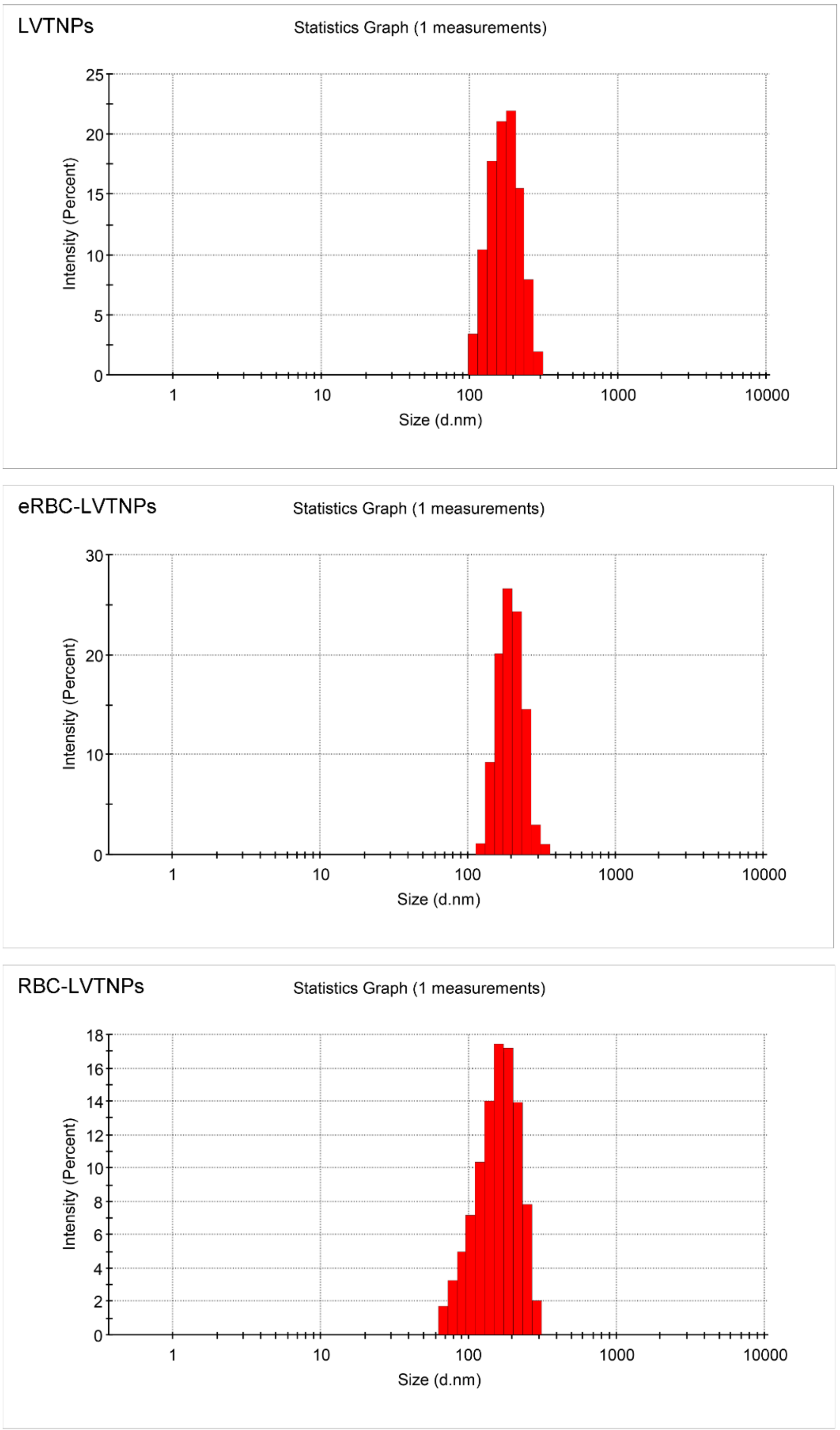

Figure S8. DLS raw data of LVTNPs, eRBC-LVTNPs, and RBC-LVTNPs (n = 3).

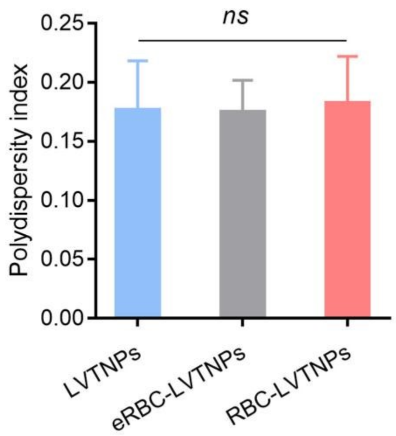

Figure S9. Polydispersity index of LVTNPs, eRBC-LVTNPs and RBC-LVTNPs (n = 3). Significance was indicated as no significance (*ns*).

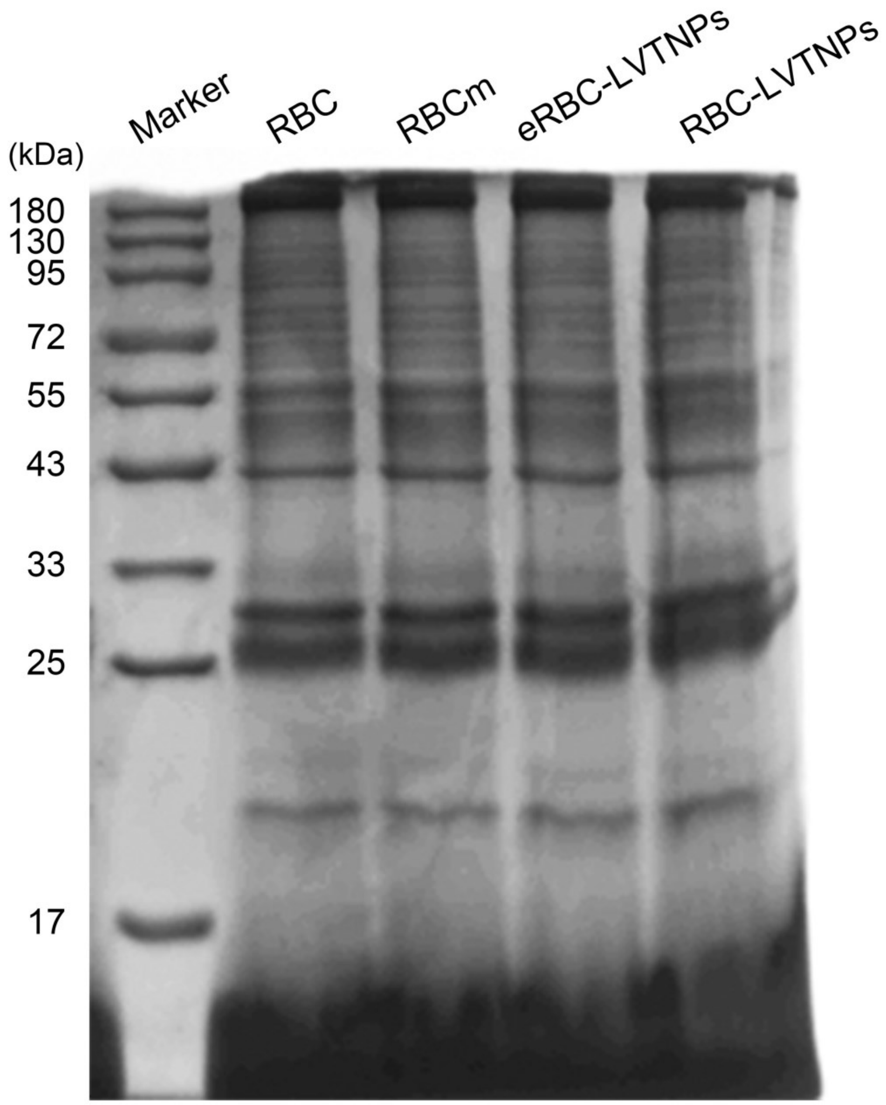

Figure S10. Proteins in RBC, RBC membrane, eRBC-LVTNPs and RBC-LVTNPs were characterized by SDS-PAGE.

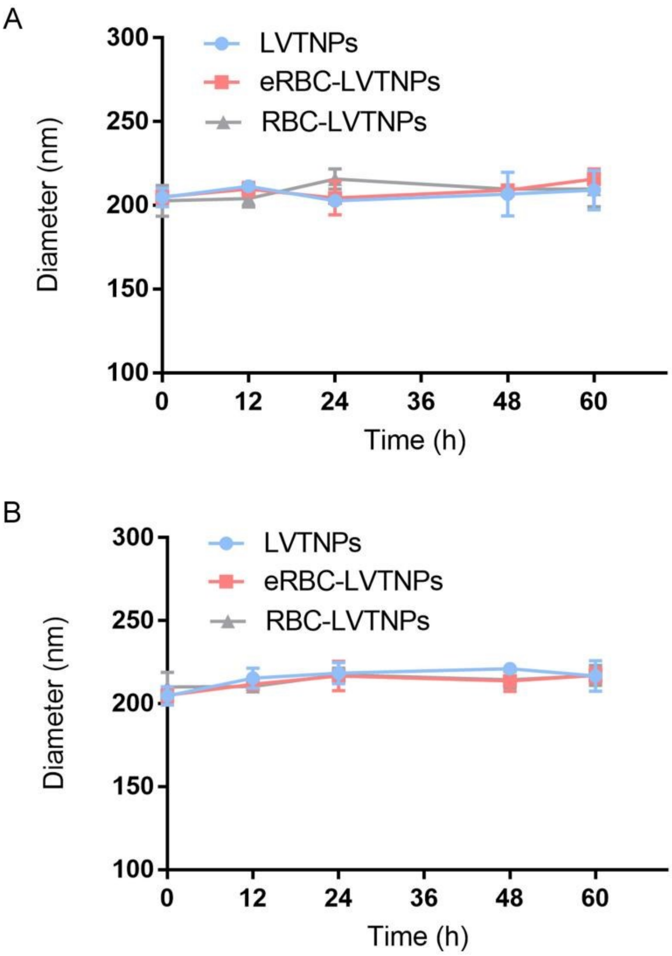

Figure S11. The stability of LVTNPs, eRBC-LVTNPs, and RBC-LVTNPs within 60 h in (A) PBS and (B) 10% serum (*n* = 3).

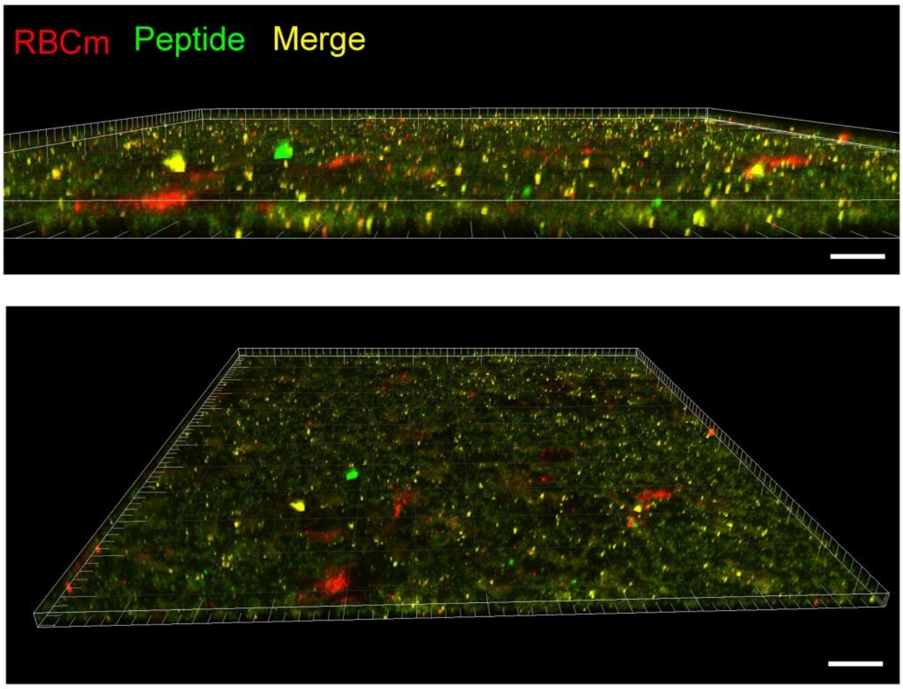

Figure S12. Representative confocal images of co-localization of RBC membrane and P4.2 peptide were used for Imaris 3D rendering (scale bar: 40 μm).

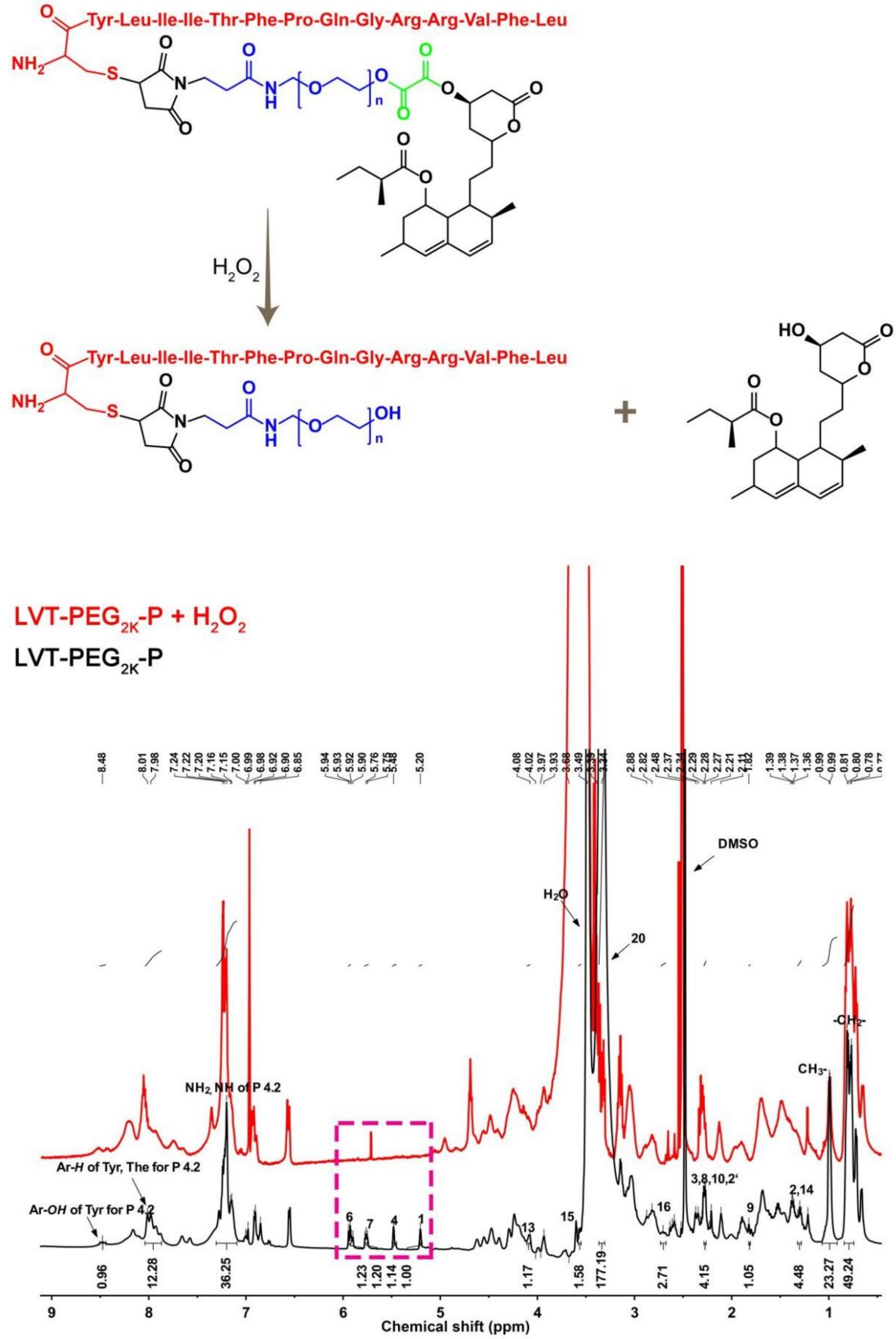

Figure S13. ^{1}H NMR spectrum of LVT-PEG$_{2K}$-P with or without H_2O_2, the drug release before and after H_2O_2 treatment was marked with a red dotted box.

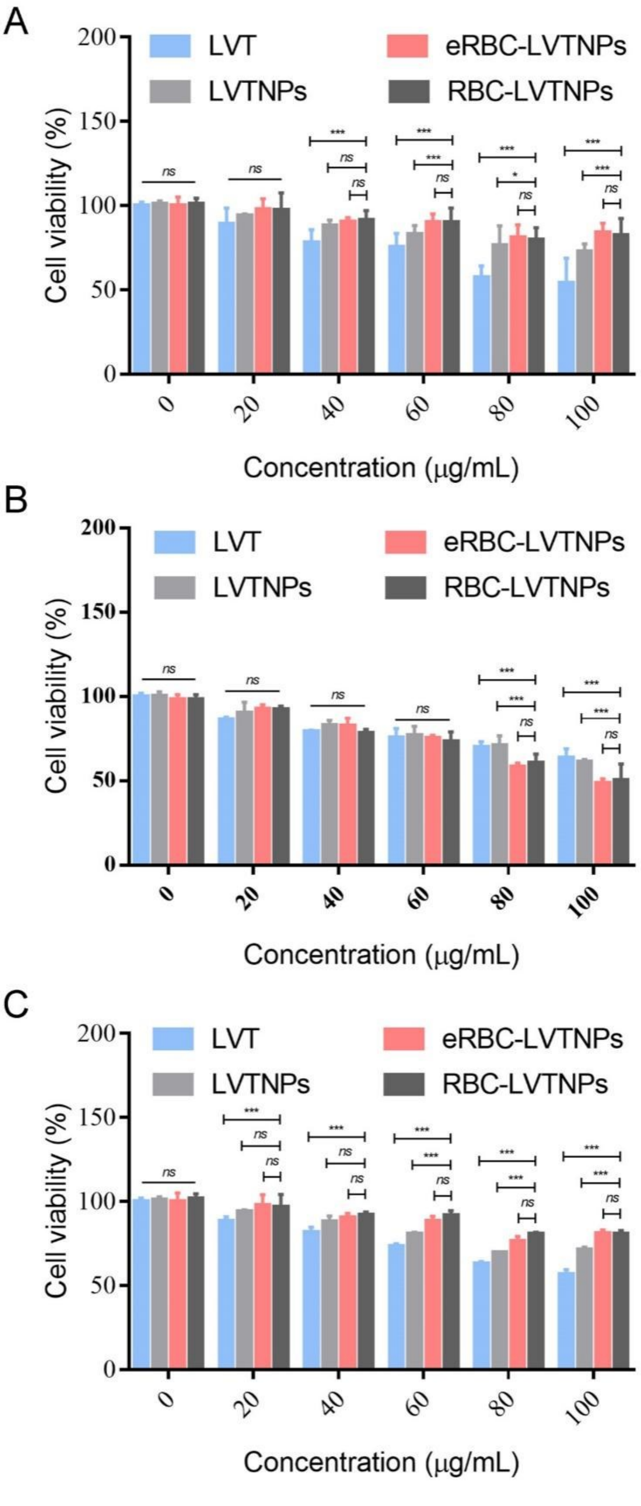

Figure S14. Cell viability of (A) ECs, (B) SMCs and (C) RAW264.7 cells after incubation with various doses of free LVT, LVTNPs, eRBC-LVTNPs, and RBC-LVTNPs for 24 h (n = 3). Significance was indicated as no significance (*ns*), $P < 0.05$ (*), or $P < 0.001$ (***).

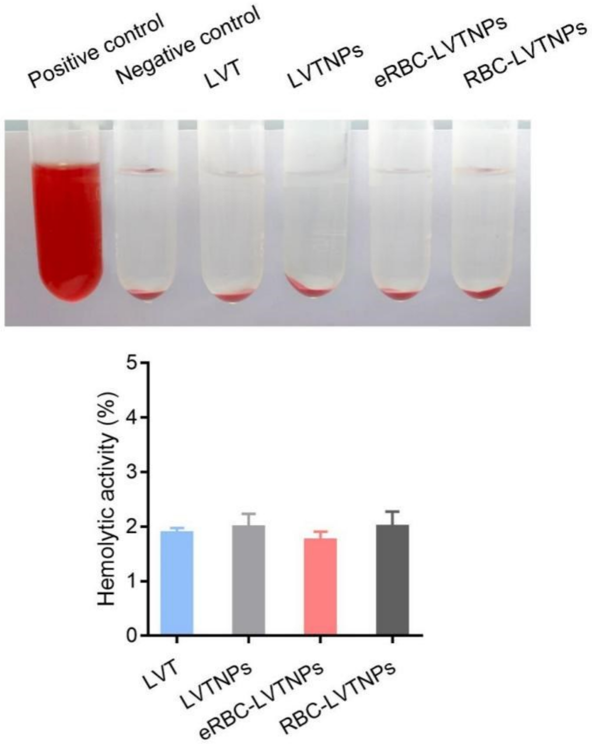

Figure S15. Images of the hemolysis test with free LVT, LVTNPs, eRBC-LVTNPs, and RBC-LVTNPs and quantification analysis of the absorbance of free LVT, LVTNPs, eRBC-LVTNPs, and RBC-LVTNPs measured at 545 nm (n = 3).

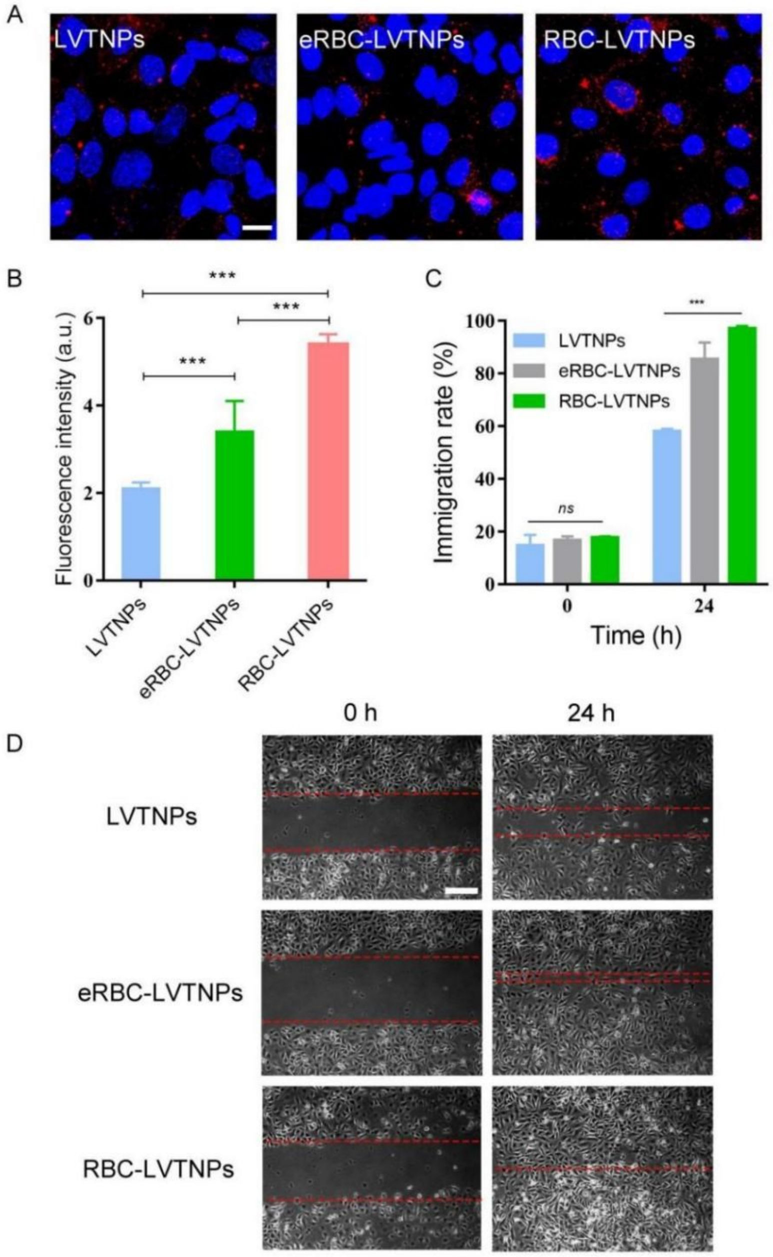

Figure S16. (A) Representative confocal images of cellular uptake of LVTNPs, eRBC-LVTNPs and RBC-LVTNPs by ECs (n = 3, scale bar: 50 μm). (B) quantification analysis of the cellular uptake of LVTNPs, eRBC-LVTNPs and RBC-LVTNPs. (C-D) Photographs and quantification analysis of ECs migration treated with LVTNPs, eRBC-LVTNPs and RBC-LVTNPs at 0 and 24 h (n = 5, scale bar: 200 μm). respectively. Significance was indicated as no significance (*ns*), or $P < 0.001$ (***).

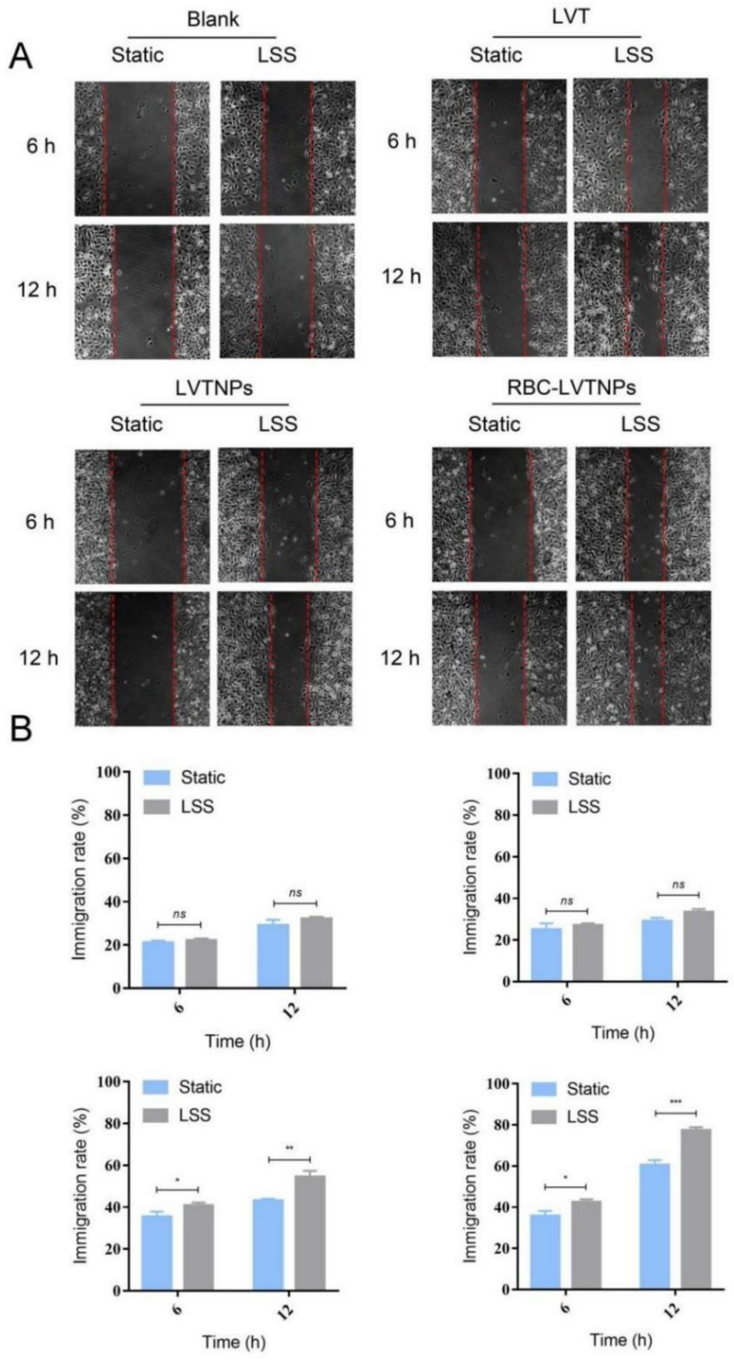

Figure S17. Photographs of ECs migration treated with saline, free LVT, LVTNPs, and RBC-LVTNPs at 6 and 12 h (A). (B) Quantification analysis of ECs migration treated with saline, free LVT, LVTNPs, and RBC-LVTNPs at 6 and 12 h, respectively (n = 5). Significance was indicated as no significance (*ns*), $P < 0.05$ (*), $P < 0.01$ (**), or $P < 0.001$ (***).

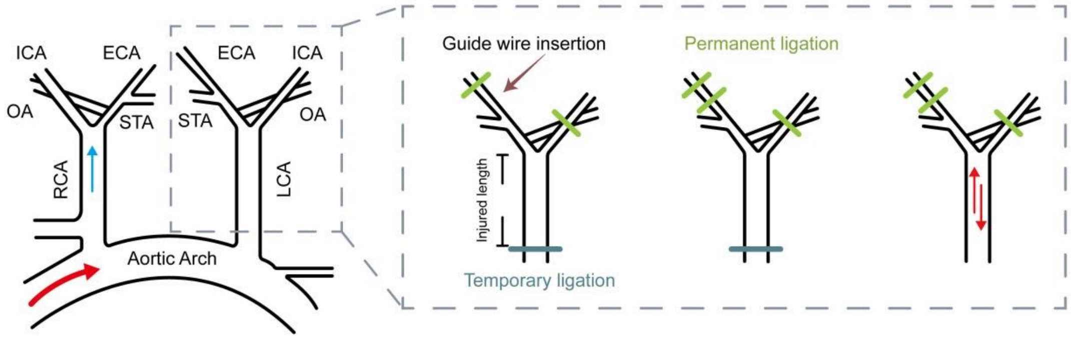

Figure S18. Schematic of guide-wire injury model establishment. The flow at ligated part of the LCA was disturbed flow with LSS.

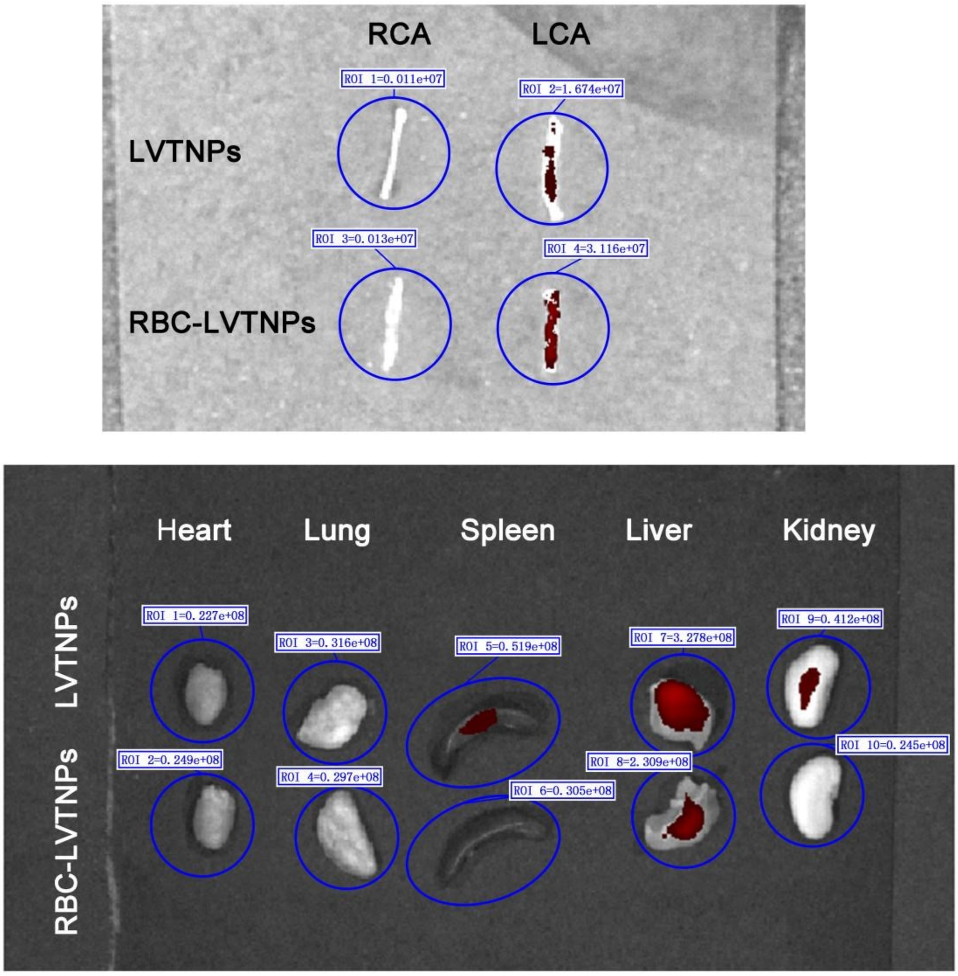

Figure S19. Original data of *ex vivo* fluorescence imaging.

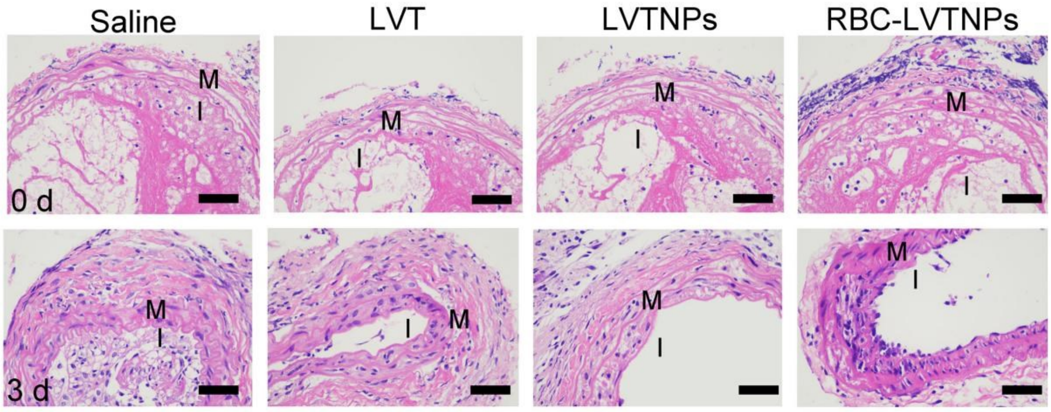

Figure S20. H&E staining of carotid artery sections from mouse model after different treatments for 0 and 3 days, “I” refers to the intima and “M” refers to the media. Images were of 200× magnification (n = 8, scale bar: 50 μm).

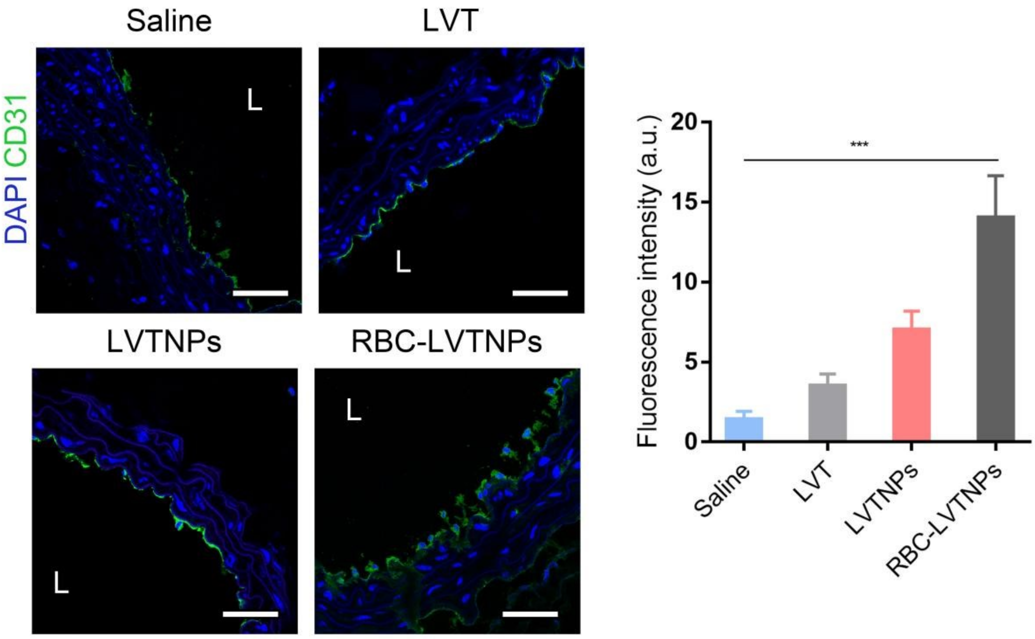

Figure S21. Immunofluorescence analyses of aorta section in *apoE*$^{-/-}$ mice and quantification analysis of the fluorescence intensity of CD31. L refers to lumen (n = 3, scale bar: 20 μm).

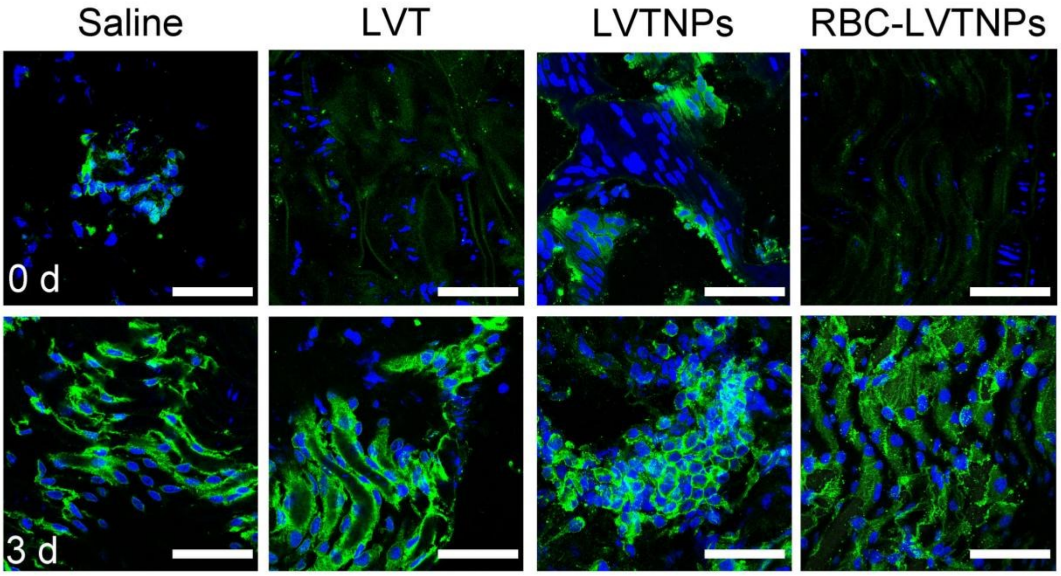

Figure S22. *En face* immunofluorescence images of LCA after different treatments at 0 and 5 days (green: CD31, n = 8, scale bar: 50 μm).

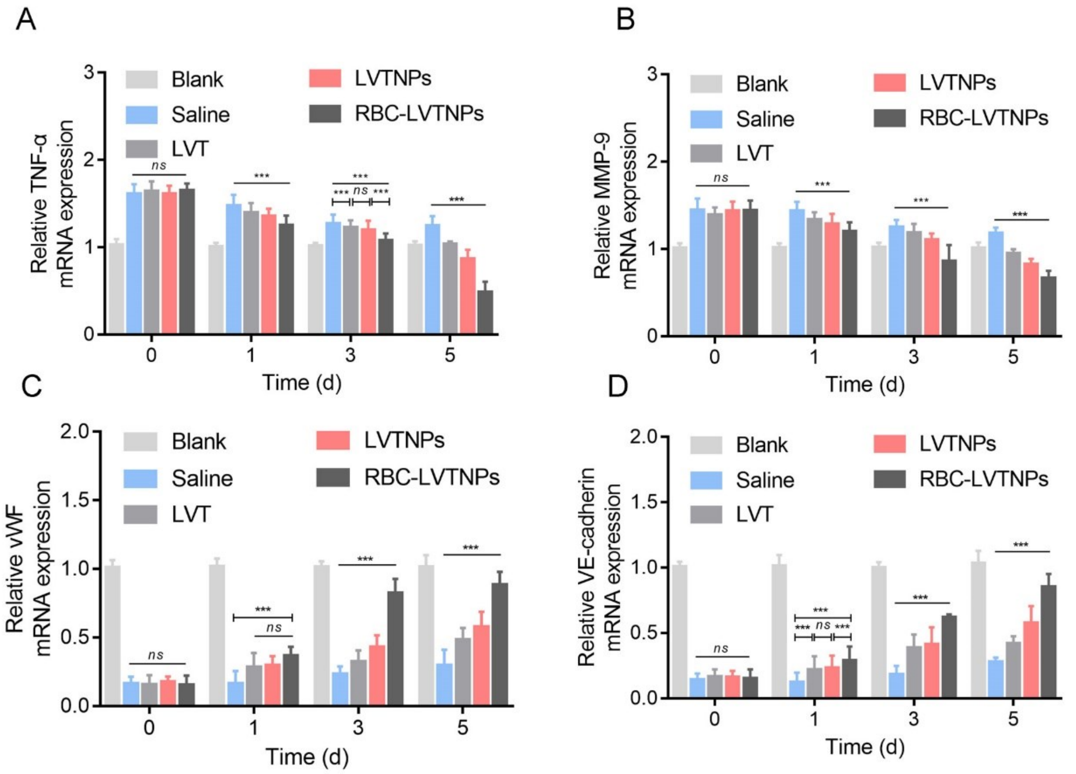

Figure S23. Quantification analysis of the mRNA expression level of (A) TNF-α, (B) MMP-9, (C) vWF, and (D) VE-cadherin (n = 15). Significance was indicated as no significance (*ns*), or $P < 0.001$ (***).

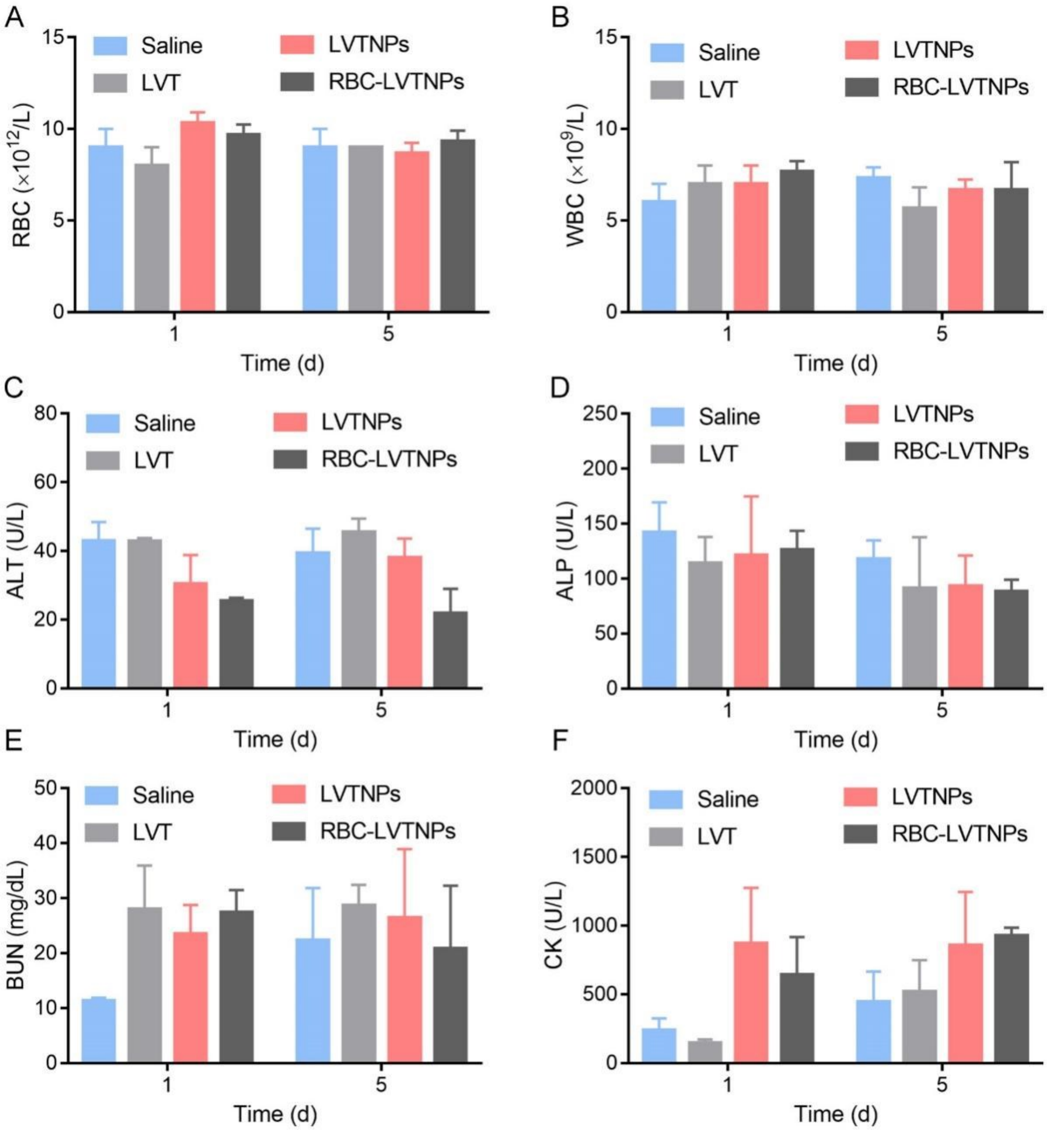

Figure S24. The RBC, WBC, ALP, ALT, BUN, and CK analysis of blood or serum from mice after various treatments for 1 and 5 days (*n* = 5).

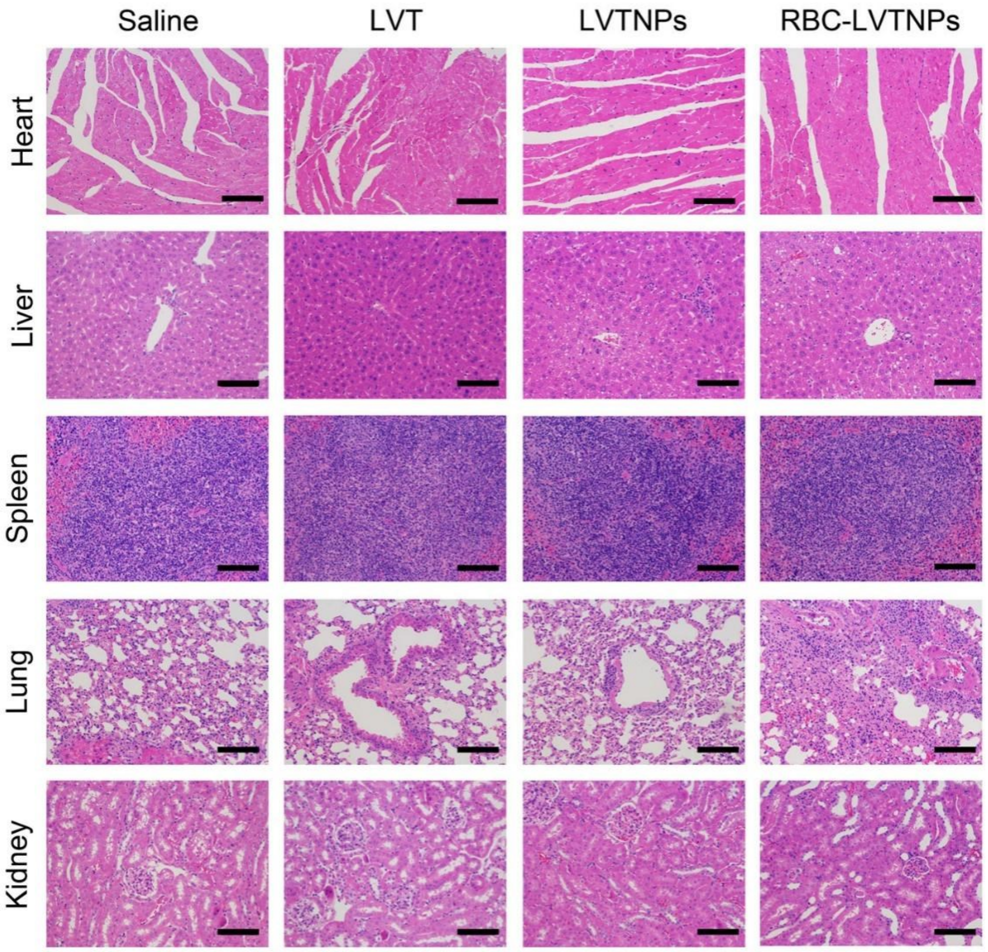

Figure S25. H&E stained sections of major organs resected from mice subjected to treatment with various formulations for 5 days (n = 5, scale bar: 100 μm).

Table S1. ^{13}C chemical shift of LVT-OC.

δ (ppm)	Atom	Ingredient
177.04	C(1')	Lovastatin
171.02	C(17)	
160.30	C(21)	Oxalyl chloride
158.30	C(21)	
133.07	C(7)	Lovastatin
131.56	C(5)	
129.56	C(4)	
128.28	C(6)	
76.59	C(13)	
67.99	C(1)	
62.33	C(15)	
41.49	C(2')	
37.24	C(9)	
36.55	C(16/10)	
35.96	C(14)	
32.93	C(2)	
32.61	C(12)	
30.64	C(8)	
27.41	C(3')	
26.8	C(3)	
24.24	C(11)	
22.82	C(19)	
16.23	C(4)	
13.86	C(16)	
11.72	C(5')	

Table S2. ^{13}C chemical shift of LVT-PEG_{2K}-P.

δ (ppm)	Atom	Ingredient
175.48	C(1')	Lovastatin
170.13	C(17)	
137.65	C(7)	
133	C(5)	
130	C(4)	
131.48	Ar	P4.2
129.22		
129.1		
127.97		
128.2	C(6)	Lovastatin
77.81	C(13)	
69.75	-CH_2CH_2O-	MPEG
68.95	C(1)	Lovastatin
60.19	C(15)	
40.88	C(2')	
38.5	C(9)	
36.92	C(16/10)	
34.96	C(14)	
32.87	C(2)	
31.89	C(12)	
31.31	C(8)	
30.08	C(3')	
28.82	C(3)	
22.56	C(11)	
21.34	C(19)	
12.33	C(4)	
11.2	C(16)	
10.73	C(5')	

Table S3. Primer sequence list for qPCR.

eNOS	Forward	CAAGGACATATGTTTGTCTGCG
	Reverse	GGTGACAAGCCGCATAC
VE-cadherin	Forward	TGGCCAAAGACCCTGACAAG
	Reverse	AGGGTTACCCCGAGAATCCA
CD31	Forward	ACCAAGGGGAGTTGTTGGAC
	Reverse	TCACTCTCCTCGGCGATCTT
vWF	Forward	CTCCCGTGCCTACCTTGAG
	Reverse	GATATGGGAGGCTTCAGGGC
VCAM-1	Forward	TGACATCTCCCCCGGATCTC
	Reverse	ACAGAGCTCAACACAAGCGT
MMP-9	Forward	AAACCCTGTGTGTTCCCGTT
	Reverse	GAAGACGCACAGCTCTCCTG
TNF-α	Forward	CCTCACACTCACAAACCACCA
	Reverse	ACAAGGTACAACCCATCGGC
α-SMA	Forward	GCCTCTGGACGTACAACTGG
	Reverse	CGGCAGTAGTCACGAAGGAA
